# Composition and assembly of the microbial community within the deep chlorophyll maximum layer of the seamount and the adjacent coastal waters in the South China Sea

**DOI:** 10.1128/msphere.00162-26

**Published:** 2026-06-03

**Authors:** Liyuan Ren, Yanpeng Shang, Zhangxian Xie, Dongxu Li, Cheng Xue, Dazhi Wang

**Affiliations:** 1 State Key Laboratory of Marine Environmental Science/College of the Environment and Ecology, Xiamen University 554979 , Xiamen, China; 2 School of Resources and Environmental Sciences, Key Laboratory of Rural Environmental Remediation and Waste Recycling, Quanzhou Normal University 117823, Quanzhou, China; Clemson University, Clemson, South Carolina, USA

**Keywords:** seamount, deep chlorophyll maxima, microbial community, co-occurrence network, deterministic and stochastic processes

## Abstract

Seamounts are generally recognized as hotspots of microbial biodiversity in the ocean, and their topography and hydrography around the seamounts significantly influence microbial biogeography. Nevertheless, the biodiversity and assembly mechanisms of microbial communities in seamount regions remain poorly understood. In this study, we investigated the diversity, composition, structure, and assembly mechanism of both large-fraction (LF; 1.6–200 μm) and small-fraction (SF; 0.2–1.6 μm) bacterial and protistan communities within the deep chlorophyll maximum (DCM) layer of the Xianbei seamount and adjacent coastal waters in the South China Sea, using 16S and 18S rDNA high-throughput sequencing. The results showed that the seamount served as a hotspot for the diversity of LF bacteria and protists in the DCM layer, which was associated with chlorophyll *a* (Chl-*a*) fluorescence in the seamount region. Microbial community composition differed between the seamount and coastal regions, with metabolically flexible heterotrophic bacteria (Pseudomonadales, Planctomycetales, and the BD2-11 terrestrial group) and broad-spectrum phagotrophic flagellates (Telonemia) being enriched in the seamount. Furthermore, co-occurrence networks in the seamount exhibited lower clustering and robustness than those in coastal waters. Model analyses revealed that stochastic processes exerted a more pronounced influence on the assembly of microbial communities. Our study revealed distinct features of microbial diversity and community structure between the seamount and adjacent coastal areas, highlighting the significance of stochastic processes in structuring microbial communities in the seamount.

**IMPORTANCE:**

Seamounts represent potential hotspots of microbial diversity, characterized by a wide array of unique microbial communities. Nevertheless, our understanding of the microbiology of seamounts remains limited. In this study, we show that microbial diversity and community structure within the deep chlorophyll maximum in the Xianbei seamount differ distinctly from those in adjacent coastal waters. We suggest that these differences are likely associated with enhanced primary production and dynamic environmental heterogeneity driven by seamount topography. Furthermore, in comparison with previous studies, our findings reveal that microorganisms of varying sizes and lifestyles display different responses to seamount effects, with particularly pronounced responses among particle-associated bacteria and protists. The findings of this study advance our understanding of microbial diversity and assembly in seamounts and provide a valuable reference for further exploration of microbial biodiversity in this region.

## INTRODUCTION

Seamounts are generally defined as undersea topographic structures exceeding a height of 1,000 m (1) and represent distinctive marine environments with considerably enhanced currents and hydrodynamic variations (2, 3). The interaction between the topography and hydrography around seamounts exerts a substantial influence on physical oceanographic processes, enhancing vertical mixing, internal waves, and Taylor columns (4). These processes create unique plankton habitats by increasing the vertical flux of nutrient-rich waters, thereby enhancing primary productivity and supporting high biodiversity, particularly around seamounts in oligotrophic oceans, a phenomenon referred to as the “seamount effects” (5, 6). For instance, an increase in plankton biomass has been observed at the C4 Seamount in the tropical western Pacific, with the physical, chemical, and biological coupling processes of the C4 Seamount corroborating the classic hypothesis of the seamount effect (7). Investigations of the Marsili and Palinuro Seamounts in the Tyrrhenian Sea revealed that the abundance and biomass of prokaryotes in the seamount sediments were significantly higher than those in the non-seamount areas and that the bacterial community structures differed between these environments (8).

Bacteria are the most prevalent microorganisms in marine environments and can consume approximately half of the daily primary production in the upper ocean (9). These microorganisms are typically categorized into free-living (FL) and particle-associated (PA) bacteria through size filtration, and they can dynamically transition between these two forms (10). Protists, a diverse and complex group of primarily unicellular eukaryotic organisms, play a vital role in the microbial food web, occupying various ecological niches as primary producers, predators, symbionts, and parasites (11, 12). Furthermore, protists and bacteria engage in symbiotic relationships that span a functional spectrum ranging from facultative to obligate and mutualistic to parasitic (13, 14). Despite their ecological significance, studies focusing on microbial ecology and the underlying mechanisms governing microbial assembly in seamount ecosystems remain scarce. This gap is especially evident regarding the differential responses of microorganisms with distinct cell sizes and trophic strategies, such as FL and PA taxa (15, 16).

Co-occurrence analysis infers putative inter-taxa relationships by examining non-random abundance covariation across samples and visualizes these relationships as networks consisting of nodes (representing microbial taxa) and edges (indicating statistically supported associations). Topological features of these networks, including complexity, modularity, keystone taxa, and robustness, provide insights into the structure of potential microbial interactions (17, 18). Such network-based approaches have not only clarified the drivers of microbial co-occurrence patterns and the response of community complexity and stability to environmental variability but also uncovered how microbial interactions modulate ecosystem functioning (19–21). However, the impact of seamounts on the complexity and stability of microbial interactions remains poorly understood.

Deep chlorophyll maxima (DCMs) are persistent features in extensive regions of oligotrophic tropical and subtropical oceans, characterized by high biomass and high biodiversity (22). Despite their significant contribution to marine primary production, microbial biodiversity, and community structures within DCMs of seamounts remain poorly understood. The Xianbei seamount, situated in the central region of the South China Sea (SCS), has a height of 3,786 m, with a peak located 208 m below the sea surface (23). As one of the largest seamounts proximal to the euphotic zone, it presents an ideal natural setting for investigating the influence of seamounts on microbial diversity and distribution (24, 25). Based on previous studies, we hypothesize that microbial biodiversity and community structure in the DCM of the Xianbei seamount region differ from those in adjacent coastal waters. To test this hypothesis, we employed a size fractionation strategy to collect both large-fraction (LF; 1.6–200 μm) and small-fraction (SF; 0.2–1.6 μm) bacterial and protistan communities from the DCM layer in the seamount region of Xianbei (XB), Xisha (XS), and Dongsha (DS) in the SCS, using high-throughput sequencing of the 16S and 18S rRNA genes, respectively. LF samples contain PA bacteria, microeukaryotes, and zooplankton, while SF samples are largely dominated by FL bacteria and picoeukaryotes (26, 27). The primary objectives of this study were to (i) compare the diversity, community composition, and co-occurrence networks of bacterial and protistan communities between seamounts and adjacent coastal waters, and (ii) explore the potential effects of seamount environments on microbial community structure.

## MATERIALS AND METHODS

### Sampling and environmental parameters

**Fig 1:**
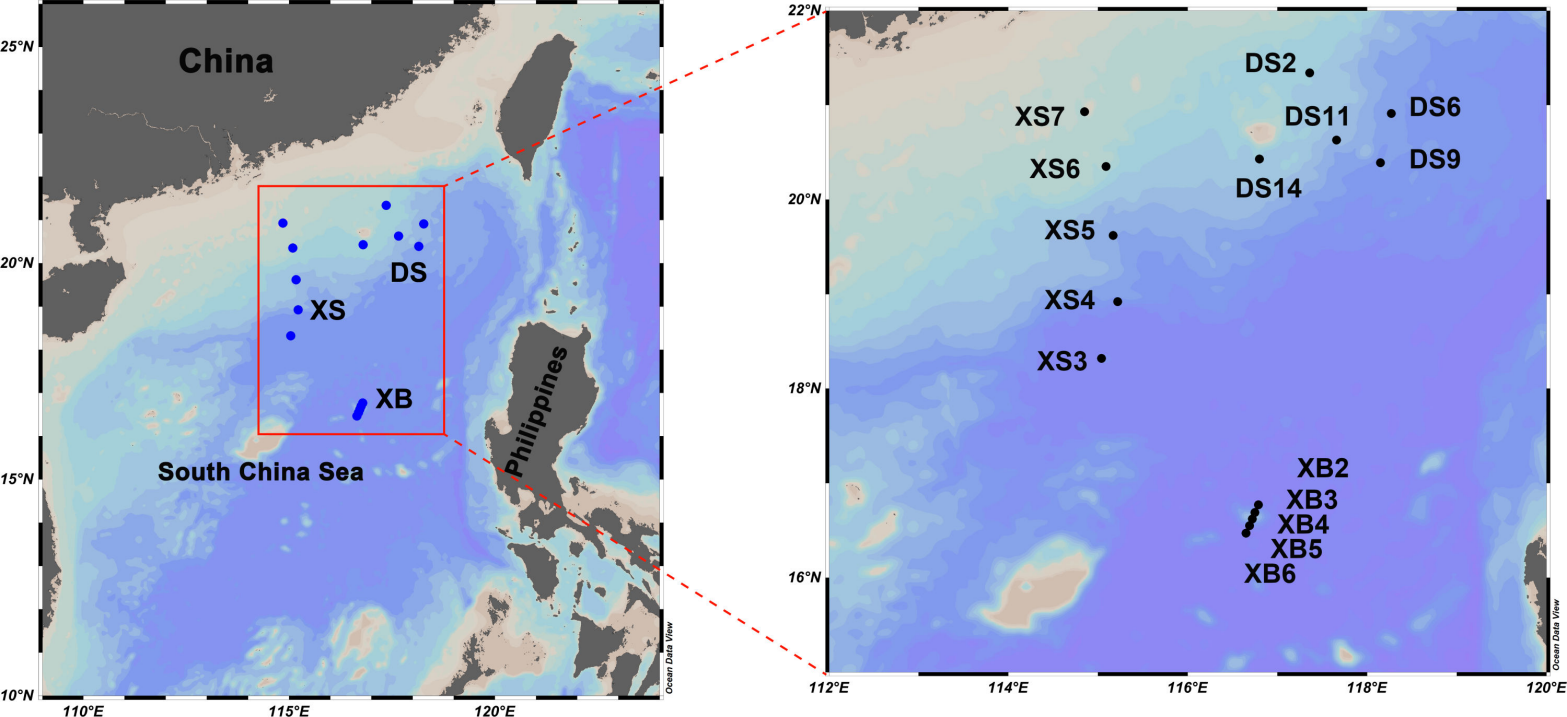
Geographic map of the sampling sites in the South China Sea. This figure was generated using Ocean Data View (ODV; odv.awi.de).

The sampling sites are located in the South China Sea, which is one of the largest marginal seas in the world with an average depth of 1,212 km (Fig. 1). The expedition, which was conducted during August and September 2021 on the research vessel “Shenkuo,” was supported by the Southern Marine Science and Engineering Guangdong Laboratory (Zhuhai). A total of 60 water samples were collected from the DCM layer at 15 sampling sites across three regions in the SCS. The precise sampling depth at each station is listed in Table S1. Sites XS3–XS7 are situated in the Xisha (XS) region, sites DS2–DS14 in the Dongsha (DS) region, and sites XB2–XB6 in the Xianbei seamount (XB) region. Seawater from the DCM layer was collected using a rosette sampler equipped with a conductivity-temperature-depth (CTD) system (Ocean Test Equipment, Inc., Fort Lauderdale, FL, USA). Each sample was pre-filtered through a 200 μm polyethylene sieve to remove large plankton and then sequentially filtered through a GF/A membrane (pore size of 1.6 μm, Waterman) and a polyethersulfone membrane (pore size of 0.2 μm, Millipore) to collect the LF and SF microbial samples, respectively. All samples were immediately frozen in liquid nitrogen and stored at −80°C for further processing. The major physicochemical characteristics, including water temperature, dissolved oxygen (DO), density, salinity, and chlorophyll *a* (Chl-*a*) fluorescence, were measured for all samples collected using the CTD system.

### DNA extraction, PCR analysis, and Illumina sequencing

For each sample, DNA was extracted from the membranes using the FastDNA SPIN extraction kit (MP Biomedicals, Santa Ana, USA) following the manufacturer’s instructions. The concentration and purity of the extracted DNA were examined using a NanoDrop2000 spectrophotometer (NanoDrop Technologies, Wilmington, DE, USA). The V4 hypervariable region of prokaryotic 16S rDNA was amplified with the 515F/806R (515F: 5′-GTGYCAGCMGCCGCGGTAA-3′/806R: 5′-GGACTACNVGGGTWTCTAAT-3′) (28, 29). The V9 hypervariable region of eukaryotic 18S rDNA was amplified with the 1380F/1510R (1380F: 5′-CCCTGCCHTTTGTACACAC-3′/1510R: 5′-CCTTCYGCAGGTTCACCTAC-3′) (30, 31). Sequencing libraries were prepared using the MetaVX Library Preparation Kit (GENEWIZ, Inc., South Plainfield, NJ), with detailed methods provided in Supplemental Methods. Next-generation sequencing was performed on an Illumina NovaSeq (PE250) platform (Illumina, San Diego, CA, USA) at Genewiz, Inc. (South Plainfield, NJ, USA).

### Data processing of amplicon sequencing data

Amplicon sequences were analyzed using the QIIME2 toolkit q2cli version 2022.2.0 (32) following the protocol. Briefly, raw Illumina NovaSeq sequencing reads were trimmed using cutadapt v3.5 to remove sequencing adaptors and PCR primers with an error rate of 0.2. Amplicon sequence variants (ASVs) were constructed separately using the DADA2 (33) algorithm implemented in the QIIME2 platform. Because the paired-end reads for the 18S rDNA amplicons did not sufficiently overlap to be merged, forward and reverse reads were truncated to 200 and 160 bp (based on quality profiles) and then concatenated for denoising using the DADA2 denoise-single command with default parameters. Subsequently, 16S rDNA ASVs were taxonomically classified using the SILVA 138 database (34), and 18S rDNA ASVs were classified using the Protist Ribosomal Reference (PR^2^) database (35). All reads classified as non-bacteria for the 16S rDNA sequencing data and non-protists for the 18S rDNA sequencing data were removed. Prior to downstream analyses, the ASVs of the bacterial and protistan communities were normalized by randomly resampling to 13,740 and 4,200 reads per sample, respectively, corresponding to the minimum sequencing depths across the bacterial and protistan data sets. Rarefaction curves for all samples are shown in Fig. S1.

### Co-occurrence network construction

We constructed co-occurrence networks in the sampling areas to identify the associations between protists and bacteria. Co-occurrence patterns were constructed using the SparCC method (36), which was implemented in the “ggClusterNet” package in R (37). To select strong interactions between microbial communities, SparCC results were filtered based on strong (*R* > 0.6) and significant (*P* < 0.05) coefficients. Network topological parameters were extracted using the “igraph” package in R. Gephi (https://gephi.org/) was used to visualize the final co-occurrence network. The relative abundance of each module in all networks was calculated from the average standardized relative abundance of the species (*z*-score) (38). The role of nodes in the network was determined by the within-module connectivity Zi and the among-module connectivity Pi, which quantify the degree of connection between a node and other nodes in its module and the degree of connection between the node and different modules, respectively (39).

### Statistical analyses

Alpha diversity indices were calculated for each sample using the QIIME2 toolkit, q2cli version 2022.2.0 (32). A comparison of alpha diversity indices was performed using a one-way ANOVA. Beta diversity was displayed using principal coordinate analysis (PCoA) based on Bray-Curtis dissimilarities, which converts data on distances between samples into map-based visualizations of those samples, facilitating the exploration of dissimilarities between samples. Analysis of similarity (ANOSIM), a robust nonparametric test for differences in resemblances among groups of samples, was employed to examine differences in the composition of microbial communities among different areas. Differential abundance analysis between seamount and coastal regions was detected as microbial biomarkers using the linear discriminant analysis (LDA) effect size (LEfSe) method, with LDA score > 3.0 and *P* < 0.05 (40). The Mantel test was performed to evaluate the linkages between the community structure of the microbial groups and environmental parameters, which were visualized using the “vegan” package. We also conducted Pearson’s correlation analysis to evaluate the relationships between environmental factors and the relative abundance of the main modules in the network.

To explore the mechanisms underlying the observed microbial succession pattern, the phylogenetic bin-based null model analysis (iCAMP) was selected to reveal the ecological drivers of microbial community assembly (41) and quantify the contribution of each ecological process to microbial community assembly. To explore the relative effects of stochastic and deterministic processes on microbial communities, we calculated Levins’ niche breadth (*B*) index for each type of microbial community (42–44). The formula was provided in the supplemental methods. The analysis was performed using the “niche.width” function within the R package “spaa.”

## RESULTS

### The physicochemical parameters of the study region

There was little spatial variation in the physical environment among the sampling sites, with temperature ranging from 22.73°C to 25.00°C, salinity from 34.12 to 34.58 PSU, density from 1,022.98 to 1,023.90 kg/m^3^, and DO from 6.27 to 6.63 mg/L (Table S1). The Chl-*a* fluorescence in the XB seamount region (0.28 ± 0.11 mg/m^3^) was lower than that in the coastal regions (XS: 0.46 ± 0.17 mg/m^3^; DS: 0.48 ± 0.15 mg/m^3^) (Table S1). The nutrient concentrations fluctuated between stations and were generally high (NH_4_–N: 3.37–12.54 μmol/L, NO_x_–N: 1.50–2.99 μmol/L, and PO_4_–P: 0.26–0.65 μmol/L) in the XS area and low in both the XB seamount (NH_4_–N: 0.03–7.36 μmol/L, NO_x_–N: 0.61–3.37 μmol/L, and PO_4_–P: 0.19–1.00 μmol/L) and DS areas (NH_4_–N: 0.37–4.10 μmol/L, NO_x_–N: 0.37–1.52 μmol/L, and PO_4_–P: 0.26–0.37 μmol/L) (Fig. S2).

### Alpha diversity of the study region

**Fig 2:**
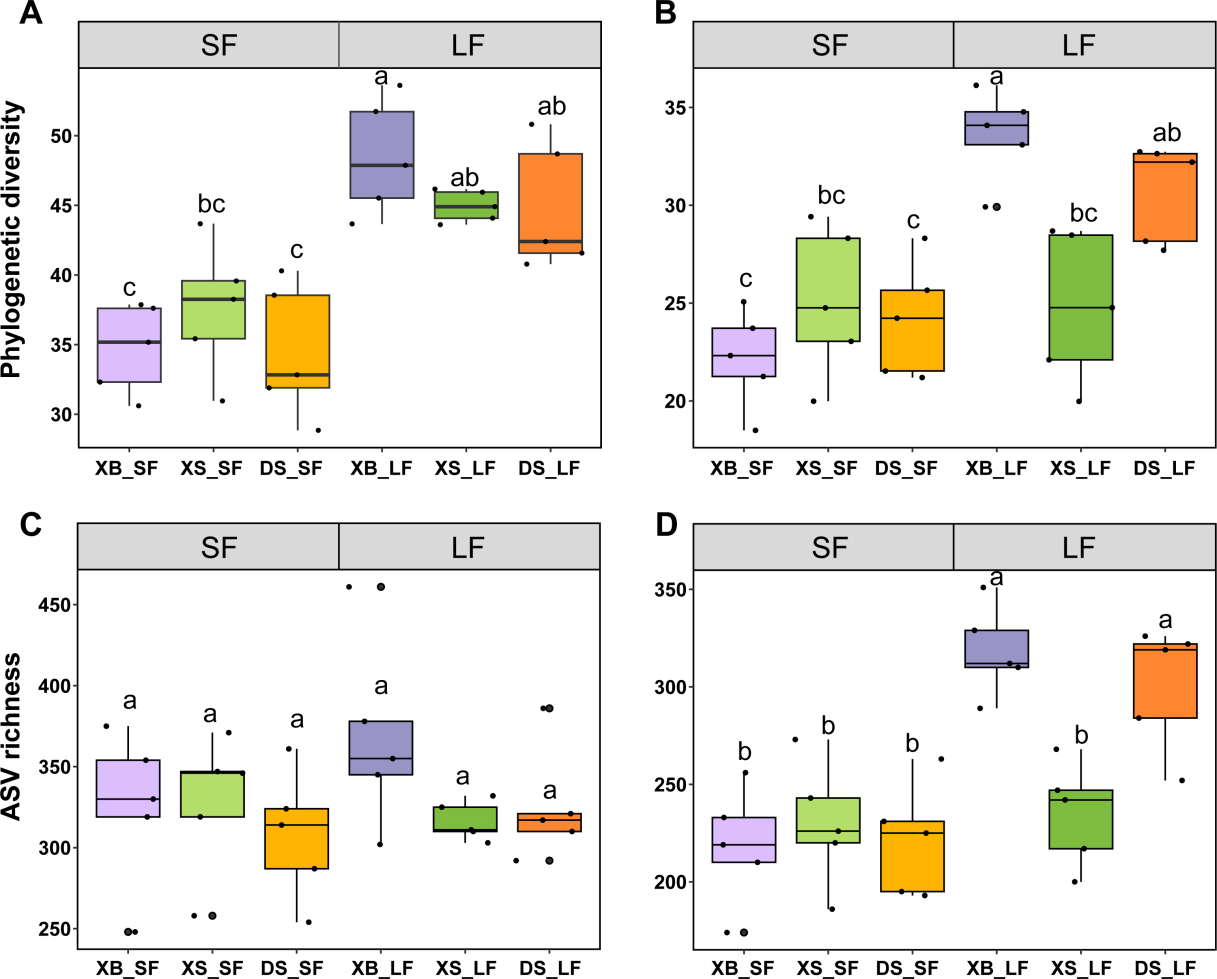
Alpha diversity indices (phylogenetic diversity [**A and B**]; ASV richness [**C and D**]) of bacteria (**A and C**) and protists (**B and D**). Different letters indicate a significant difference at the *P* < 0.05 level using one-way ANOVA.

Phylogenetic diversity and ASV richness revealed that the alpha diversity indices of the LF microbial community were generally higher in the seamount region than in the coastal region. However, these differences were not statistically significant for bacterial communities (Fig. 2). Alpha diversity indices of the SF microbial community varied insignificantly between the seamount and coastal regions. Clear differences in alpha diversity were observed between the SF and LF microbial communities (Fig. 2). The phylogenetic diversity of LF bacteria was considerably higher than that of SF bacteria in the seamount and DS coastal regions; however, the difference in ASV richness between the two fractions was insignificant (Fig. 2A and C). The phylogenetic diversity and ASV richness of the LF protistan communities in the seamount and DS coastal regions were also significantly higher than those of the SF protistan communities (Fig. 2B and D).

### Community composition of bacteria and protists

**Fig 3:**
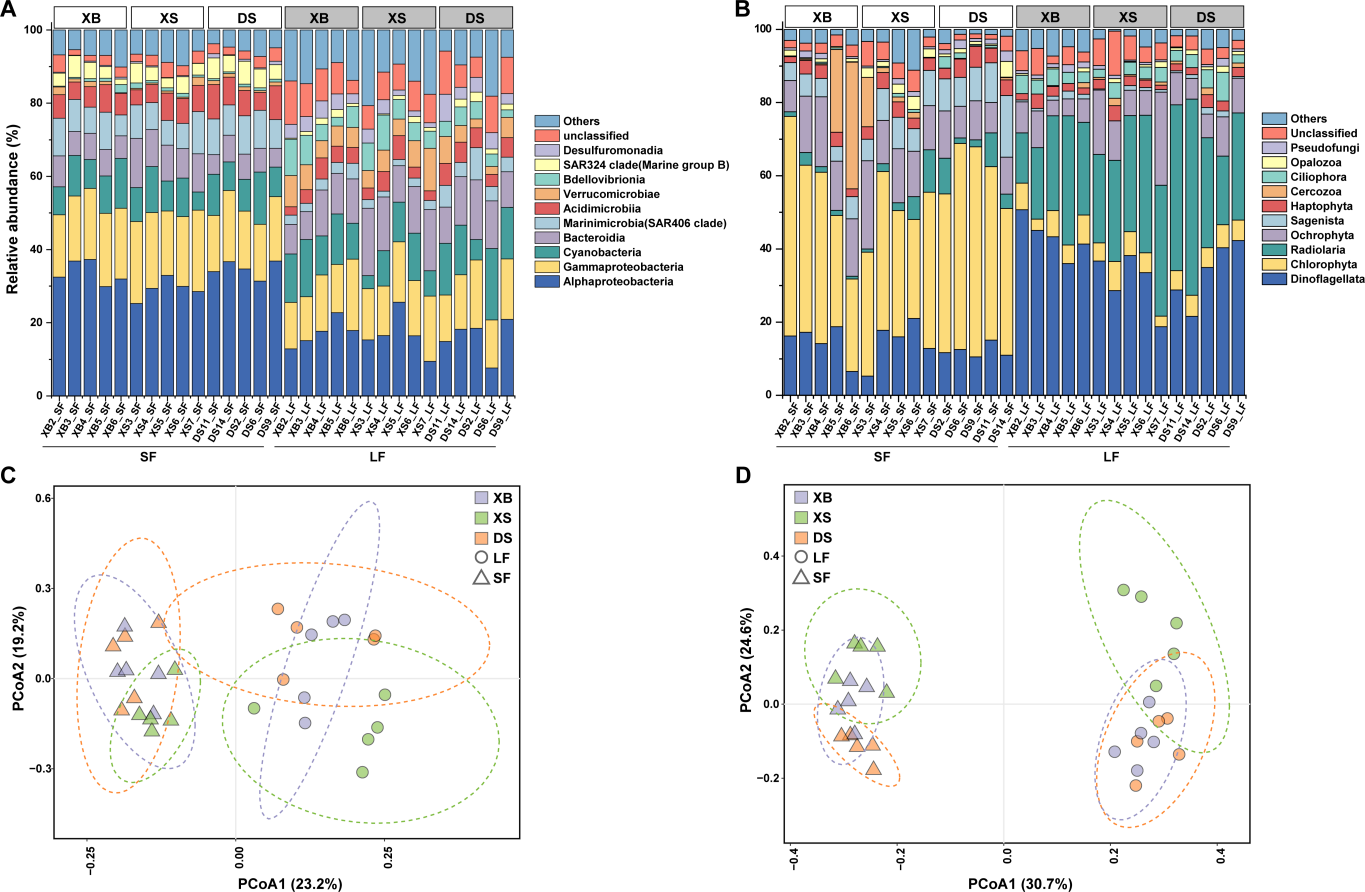
Microbial community in the DCM water of XB, XS, and DS. (**A and B**) Bar plots illustrating the bacterial (**A**) and protistan (**B**) composition. (**C and D**) Unconstrained PCoA illustrating community dissimilarities of bacteria (**C**) and protists (**D**). The difference between groups was tested using the ANOSIM test.

Different taxa in the bacterial community showed relatively constant ASV proportions between the seamount and coastal areas (Fig. 3A; Fig. S3). The dominant bacterial classes were Alphaproteobacteria and Gammaproteobacteria. They accounted for 7.6%–37.3% (SF: 25.3%–37.3%; LF: 7.6%–25.6%) and 12.0%–22.4% (SF: 15.3%–22.4%; LF: 12.0%–19.5%) of all bacteria, respectively (Fig. 3A). Differences in the taxonomic composition of bacteria were more significant between size fractions than between areas (Fig. 3C; Table S2). There were obvious fluctuations in the ASV ratios for some protist taxa (Fig. 3B; Fig. S4 and S5). Highly abundant Chlorophyta (SF: 25.2%–60.0%; LF: 2.9%–8.0%) and Dinoflagellate (SF: 5.2%–21.0%; LF: 18.8%–50.7%) largely dominated the SF and LF protistan communities, respectively. In addition, Radiolaria (SF: 0.8%–11.6%; LF: 13.7%–53.6%) also constituted a high proportion of LF protists and dominated in place of Dinoflagellate at several coastal stations (Fig. 3B), of which the main class was Polycystinea (Fig. S4). Notably, Cercozoa was only found in the seamount and XS coastal regions (Fig. 3B), with *Massisteria marina* as the dominant species (Table S3). In addition to the seven ASVs of *M. marina* shared with the XS coastal area, the seamount area had two unique ASVs (Fig. S6). We also observed significant spatial differences in protistan community composition between the seamount and coastal areas, as determined by PCoA (Fig. 3D) and ANOSIM (Table S2).

**Fig 4:**
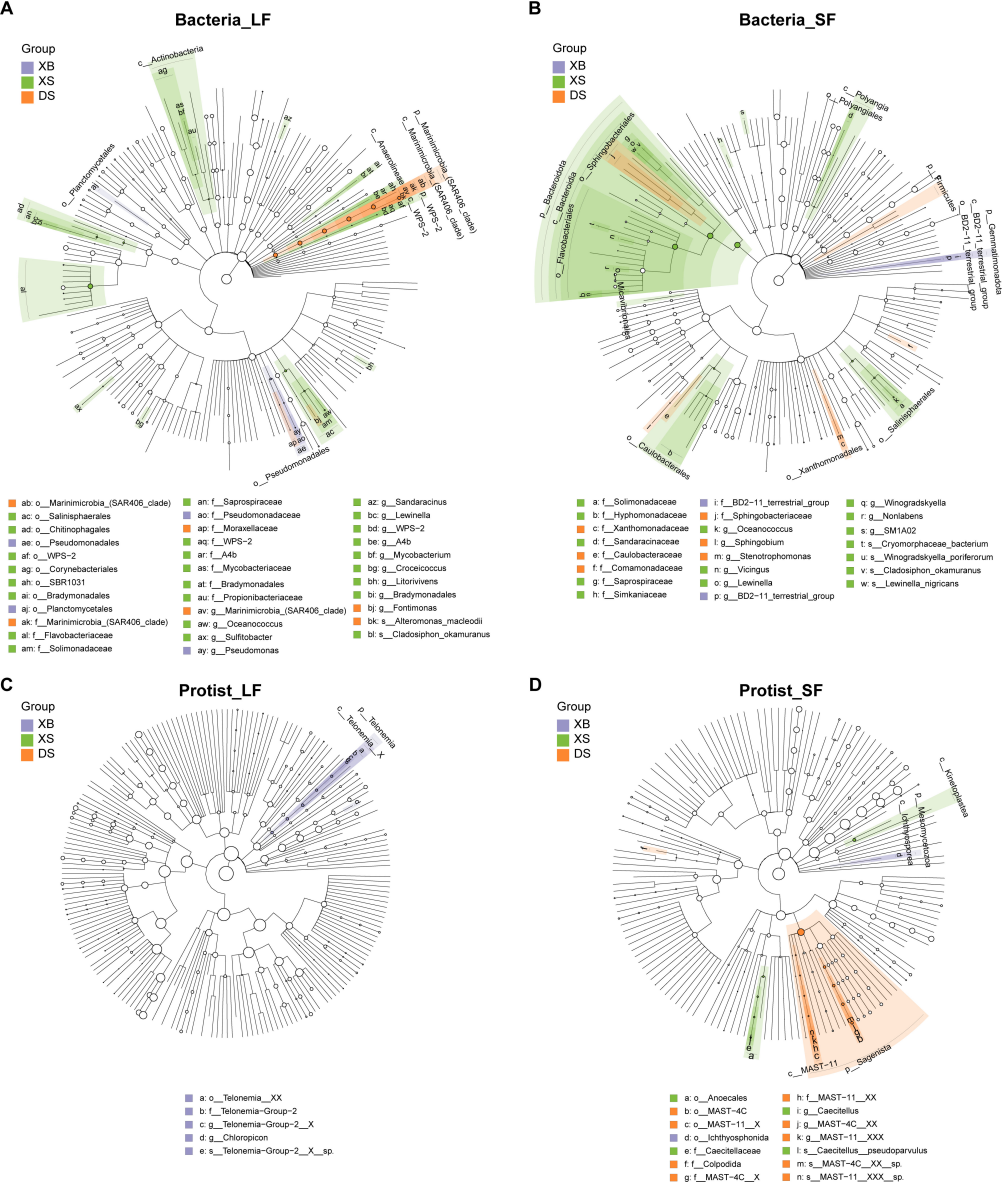
LEfSe cladogram of bacterial (**A and B**) and protistan (**C and D**) communities associated with the seamount and coastal region. Microbial lineages with LDA values of 3 or higher are displayed. Differences are represented in the color of the most abundant class. Each circle’s diameter is proportional to the taxon’s abundance. Circles represent phylogenetic levels from domain to genus, inside out.

Based on LEfSe analysis (LDA score > 3), the seamount and coastal regions harbored distinct assemblages of significantly discriminant taxa in both size fractions of bacteria and protists (Fig. 4). For the LF bacterial community (Fig. 4A), the XS coastal region exhibited the highest number of discriminant lineages, dominated by Actinobacteria-related groups (e.g., Corynebacteriales), along with Chitinophagales, Flavobacteriaceae, and Salinisphaerales, whereas LF bacteria in the DS coastal region were characterized by Marinimicrobia (SAR406 clade). Within the seamount region, Pseudomonadales (*Pseudomonas*) and Planctomycetales were identified as discriminant taxa in the LF bacteria. The SF bacterial community in the seamount region was distinguished by Gemmatimonadota (BD2-11 terrestrial group), which were consistently identified as discriminant taxa from phylum to genus levels (Fig. 4B). SF bacteria in the XS coastal region were mainly affiliated with Bacteroidota (dominated by Bacteroidia) and related families, as well as Caulobacterales and Salinisphaerales, whereas those in the DS coastal region were enriched in Firmicutes, Xanthomonadales, *Sphingobium,* and Sphingobacteriales (Fig. 4B). For protists, nearly all discriminant taxa in the LF were detected in the seamount region, among which Telonemia was identified as a discriminant group across multiple taxonomic ranks (Fig. 4C). In contrast, SF protists exhibited region-specific discriminant assemblages: Sagenista (MAST-11 and MAST-4C) in the DS coastal region, Caecitellaceae, and Kinetoplastea in the XS coastal region, and Mesomycetozoa (Ichthyosporea) in the XB seamount region (Fig. 4D).

### Co-occurrence patterns of protist-bacterium microbiota

**Fig 5:**
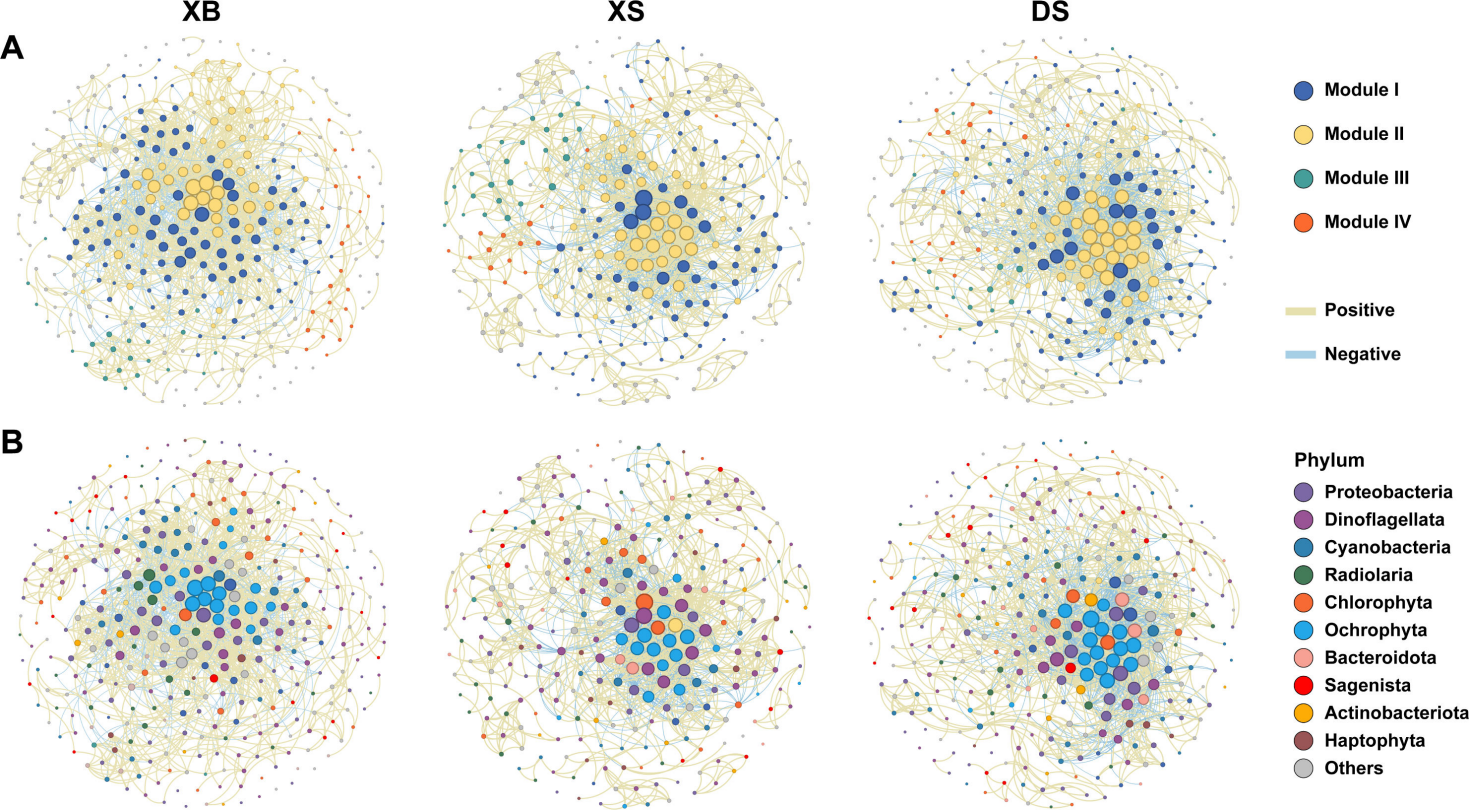
Co-occurrence patterns of protist-bacterium microbiota in XB seamount, XS, and DS areas. The nodes are colored according to modules in panel **A** and taxonomy in panel **B**. A connection represents a strong (SparCC correlation threshold *R* > |0.6|) and significant (*P* < 0.05) correlation. Positive connections are in lemon chiffon, and negative connections are in sky blue. The size of each node is proportional to the degree of the ASVs.

**Fig 6:**
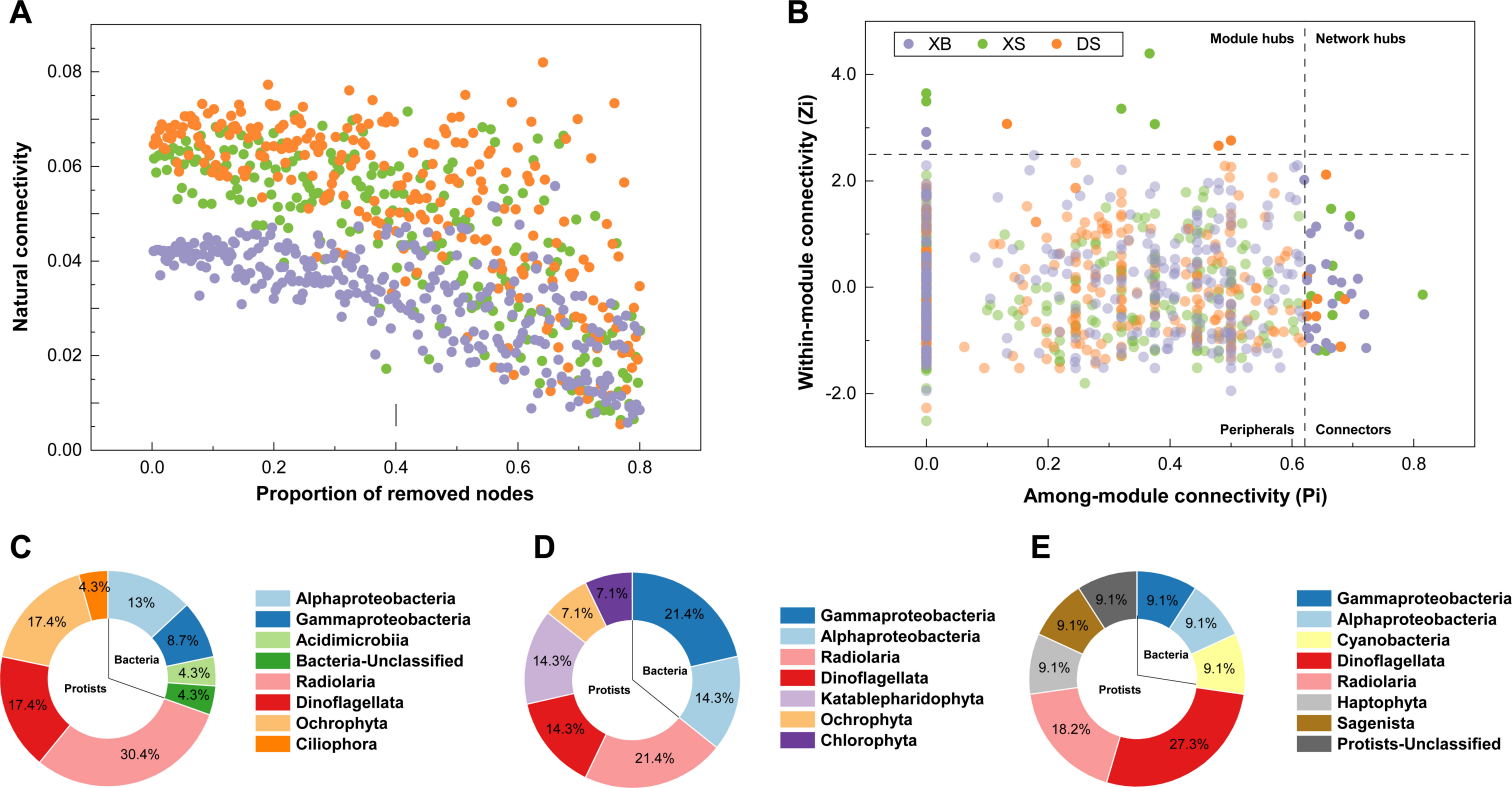
The robustness and keystone of co-occurrence patterns. (**A**) The robustness of different networks is expressed by natural connectivity after removing a certain proportion of nodes. (**B**) Node classification in networks to identify putative keystone taxa. (**C–E**) Composition of keystone taxa in networks for XB (**C**), XS (**D**), and DS (**E**).

Given the importance of biotic associations in determining community variation, we constructed co-occurrence networks of protistan and bacterial communities to further explore the effects of seamounts on biotic associations (Fig. 5). All networks exhibited scale-free properties, as all network degrees followed a power law distribution, indicating that the network structures were not random (Fig. S7). The results of the basic structure of all networks showed that the network in the seamount area differed from that in the other two coastal areas (Table 1). The number of shared edges in the seamount network was intermediate to that of the two coastal networks (Table 1). In addition, the seamount network exhibited the highest modularity and lowest average clustering coefficient (Table 1). Furthermore, the natural connectivity of the seamount network was significantly lower than that of the coastal waters, even when a small proportion of the network nodes were removed (Fig. 6A). This indicates that the network in the seamount had the lowest robustness; that is, its network structure was the most vulnerable to destruction.

**TABLE 1:** Major topological properties of the empirical and random co-occurrence patterns of protist-bacterium microbiota in XB, XS, and DS

Network properties	XB	XS	DS
Empirical networks			
Nodes	360	286	315
Edges	3,434	2,668	3,668
Positive	2,292 (66.74%)	1,886 (70.69%)	2,404 (65.54%)
Negative	1,142 (33.26)	782 (29.31%)	1,264 (34.46%)
Average degree	10.251	9.845	12.309
Modularity	0.893	0.626	0.737
Average clustering coefficient	0.431	0.538	0.496
Average path length	2.620	4.033	2.452
*R*^2^ of power-law	0.684	0.525	0.625
Random networks			
Modularity (SD)	0.003 (0.053)	0.002 (0.055)	0.003 (0.043)
Average clustering coefficient (SD)	0.031 (0.004)	0.036 (0.004)	0.041 (0.003)
Average path distance (SD)	2.742 (0.004)	2.691 (0.004)	2.544 (0.002)

Additionally, we identified possible keystone taxa in the networks by categorizing nodes into four groups (network hubs, module hubs, connectors, and peripherals; see Materials and Methods for details) based on their within-module (Zi) and among-module (Pi) values (Fig. 6B). Module hubs and connectors have been identified as keystone taxa because of their critical significance in network topology. There were 23 keystone nodes in the seamount network, 14 in the XS network, and 11 in the DS network (Fig. 6B). Members of Gammaproteobacteria (bacteria), Alphaproteobacteria (bacteria), Radiolaria (protists), and Dinoflagellata (protists) were the most prominent keystone assemblages in all networks, as they accounted for more than half of the relative abundance of keystone taxa in all networks (Fig. 6C through E). Acidimicrobiia (Actinomarinales) and Ciliophora (Spirotrichea) were the specific keystone taxa in the seamount network (Fig. 6C; Table S4). Katablepharidophyta (Katablepharidales) and Chlorophyta (Mamiellales) would be the special keystone taxa in networks of the XS coastal area (Fig. 6D; Table S4). Cyanobacteria (Synechococcales), Haptophyta (Prymnesiophyceae_Clade_D), and Sagenista (MAST-7B) were the keystone taxa in the DS coastal networks (Fig. 6E; Table S4). Notably, only the networks of the seamount and XS coastal areas shared two keystone nodes, implying that the keystone taxa were not conserved at the node level (Fig. S8).

### Environmental factors influencing microbial communities

**Fig 7:**
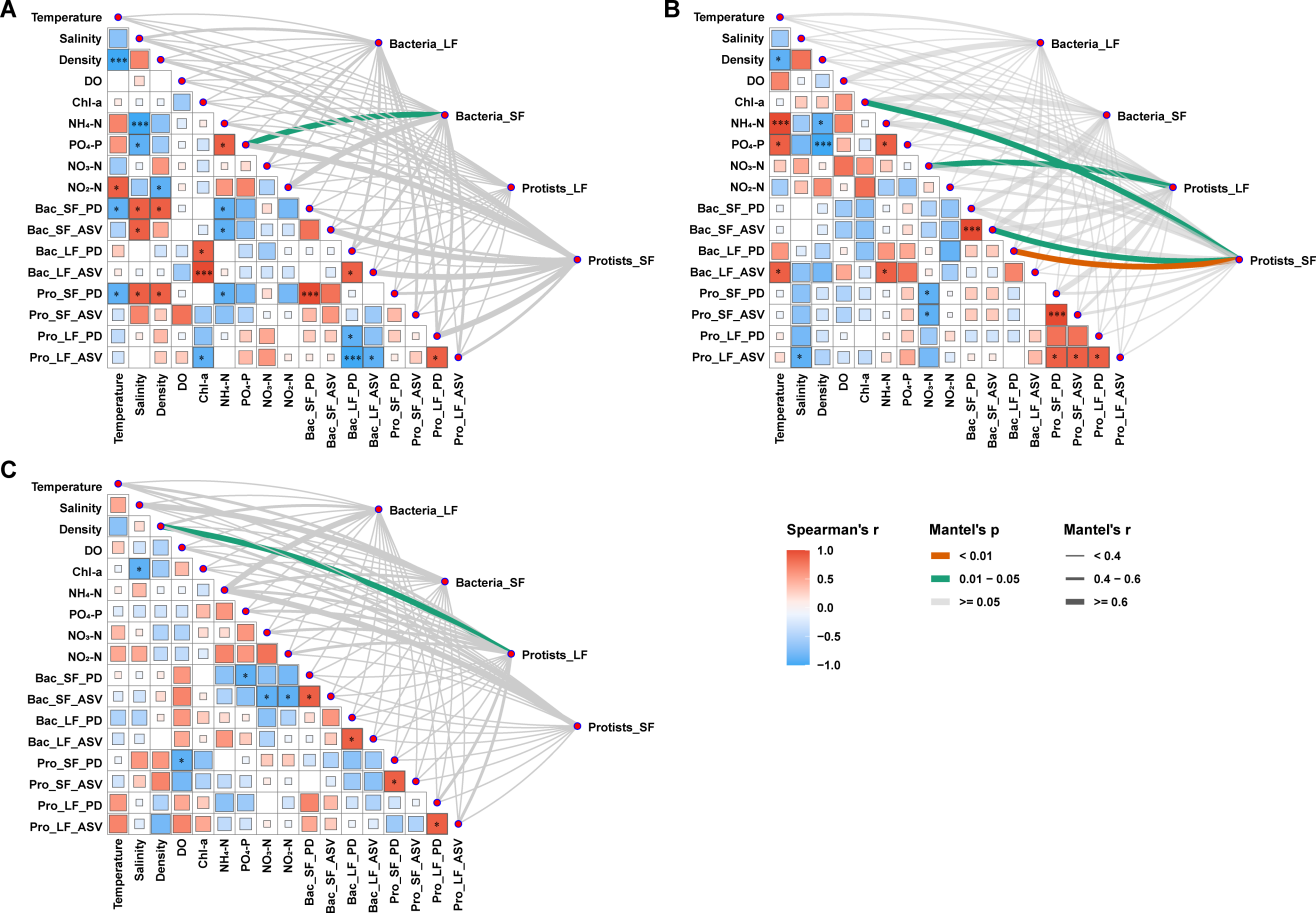
Abiotic and biotic drivers of microbial community in XB (**A**), XS (**B**), and DS (**C**) regions. Pairwise comparisons of environmental and biotic factors are shown at the lower-left, with a color gradient representing Spearman’s correlation coefficients. Microbial community composition was correlated to each environmental or biotic factor by Mantel tests. Edge width corresponds to R value and edge color indicates statistical significance. DO, dissolved oxygen; Chl-*a*, chlorophyll-*a* fluorescence; NH_4_–N, ammonium nitrogen; NO_3_–N, nitrate nitrogen; NO_2_–N, nitrite nitrogen; PO_4_–P, phosphate phosphorus; Bac_SF_PD, phylogenetic diversity of SF bacteria; Bac_SF_ASV, ASV_richness of SF bacteria; Bac_LF_PD, phylogenetic diversity of LF bacteria; Bac_LF_ASV, ASV_richness of LF bacteria; Pro_SF_PD, phylogenetic diversity of SF protists; Pro_SF_ASV, ASV richness of SF protists; Pro_LF_PD, phylogenetic diversity of LF protists; Pro_LF_ASV, ASV_richness of LF protists.

Spearman correlation analysis revealed that environmental factors significantly influenced microbial alpha diversity in the seamount region (Fig. 7). Specifically, the alpha diversity of SF microorganisms was significantly correlated with temperature, salinity, water density, and ammonium concentration, whereas that of LF microorganisms was significantly correlated with the Chl-*a* fluorescence (Fig. 7A).

To identify factors influencing community structure, Mantel tests were used to examine relationships between community composition and environmental factors. In the seamount region, phosphate concentration was significantly correlated with SF bacterial community composition (*r* = 0.631, *P* = 0.017; Fig. 7A). In the XS coastal region, nitrate concentration was significantly correlated with the LF protistan composition (*r* = 0.579, *P* = 0.042; Fig. 7B), while Chl-*a* fluorescence was significantly correlated with the SF protistan composition (*r* = 0.602, *P* = 0.050; Fig. 7B). Additionally, the ASV richness of SF bacteria (Bac_SF_ASV: *r* = 0.646, *P* = 0.050) and the phylogenetic diversity of LF bacteria (Bac_LF_PD: *r* = 0.506, *P* = 0.008) were significantly correlated with the composition of SF protists (Chl-*a*: *r* = 0.602, *P* = 0.050) in the XS coastal region (Fig. 7B). In the DS coastal region, water density (*r* = 0.790, *P* = 0.008) was significantly correlated with LF protistan composition (Fig. 7C).

**Fig 8:**
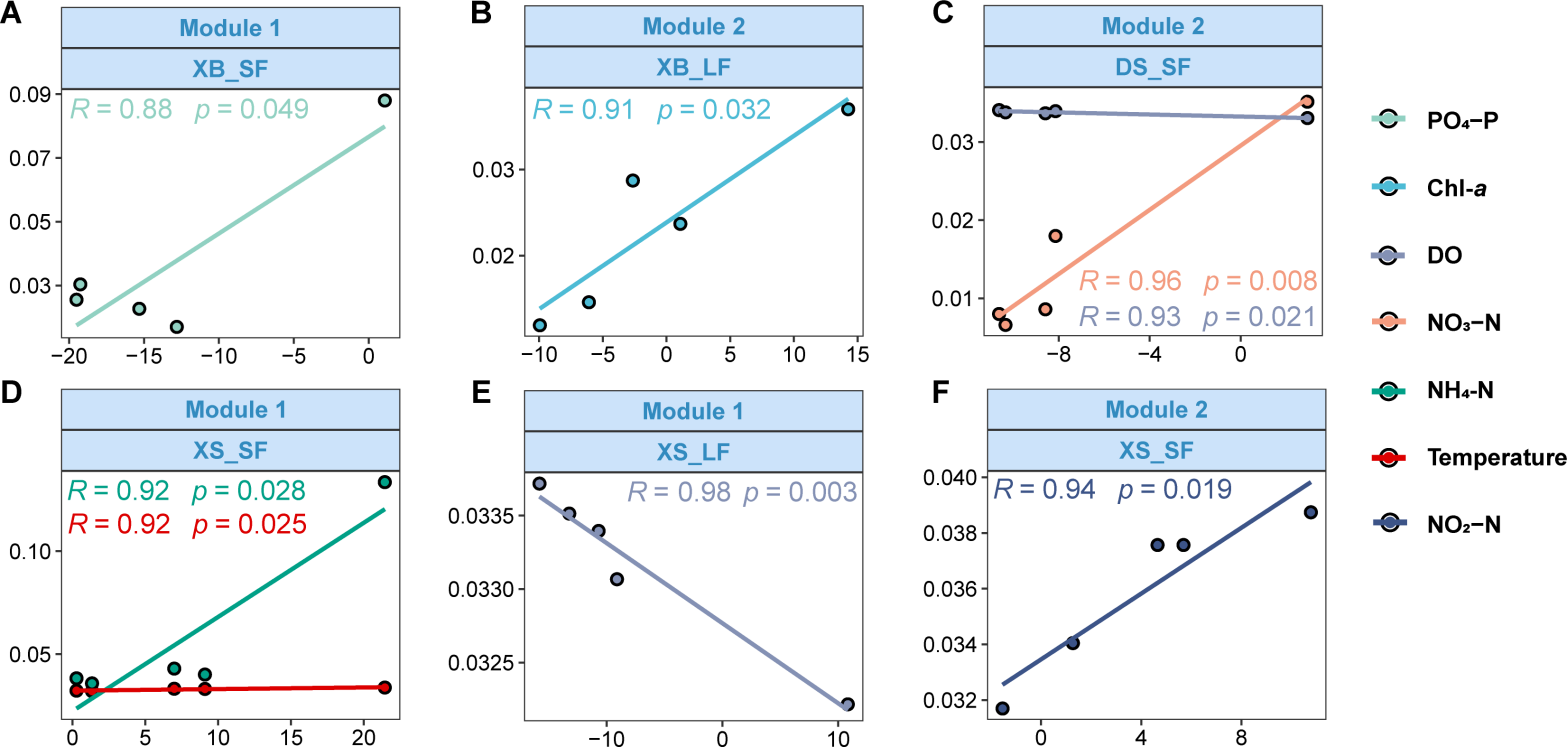
The Pearson correlation analysis of the relative abundance of main ecological clusters in the co-occurrence network and environmental factors in XB (**A and B**), DS (**C**), and XS (**D–F**) regions. DO, dissolved oxygen; Chl-*a*, chlorophyll-*a* fluorescence; NH_4_–N, ammonium nitrogen; NO_3_–N, nitrate nitrogen; NO_2_–N, nitrite nitrogen; PO_4_–P, phosphate phosphorus.

We further examined the relationships between environmental factors and the relative abundance of the top two key microbial assemblages with the highest number of nodes in each co-occurrence network (Fig. 8). Physical parameters (e.g., dissolved oxygen and temperature) and nutrient conditions (e.g., nitrate, nitrite, ammonium, and phosphate) collectively contribute to divergent microbial associations between seamount and coastal waters, with distinct key environmental drivers across the three networks. In the seamount network, Pearson correlation analysis detected a significant positive correlation between the relative abundance of SF microorganisms in Module 1 and phosphate concentration (Fig. 8A), and between the relative abundance of LF microorganisms in Module 2 and Chl-*a* fluorescence (Fig. 8B). In the DS coastal network, the relative abundance of SF microorganisms in Module 2 was significantly positively correlated with nitrate concentration and negatively correlated with dissolved oxygen (Fig. 8C). In the XS coastal network, the relative abundance of SF microorganisms in Module 1 was significantly positively correlated with ammonium concentration and temperature (Fig. 8D), that of LF microorganisms in Module 1 was significantly positively correlated with dissolved oxygen (Fig. 8E), and that of SF microorganisms in Module 2 was significantly positively correlated with nitrite concentration (Fig. 8F). The taxonomic composition of these dominant modules differed substantially between the seamount and coastal regions (Fig. S10).

### Community assembly mechanisms of microbial communities

We first examined whether microbial communities exhibited a distance–decay relationship (DDR) across the gradient from seamount to coastal waters. No obvious DDR was observed between community similarity and geographic distance for either bacteria or protists (Fig. S9). Although the DDR was significant for SF protists (*R^2^* = 0.048, *P* = 0.014), the low coefficient of determination (*R^2^* < 0.1) indicated only a weak decay in community similarity with increasing geographic distance (Fig. S9). These results suggest that linear geographic distance explains only a small proportion of β-diversity at the spatial scale of this study, and that community turnover does not conform to a simple isolation-by-distance pattern.

**Fig 9:**
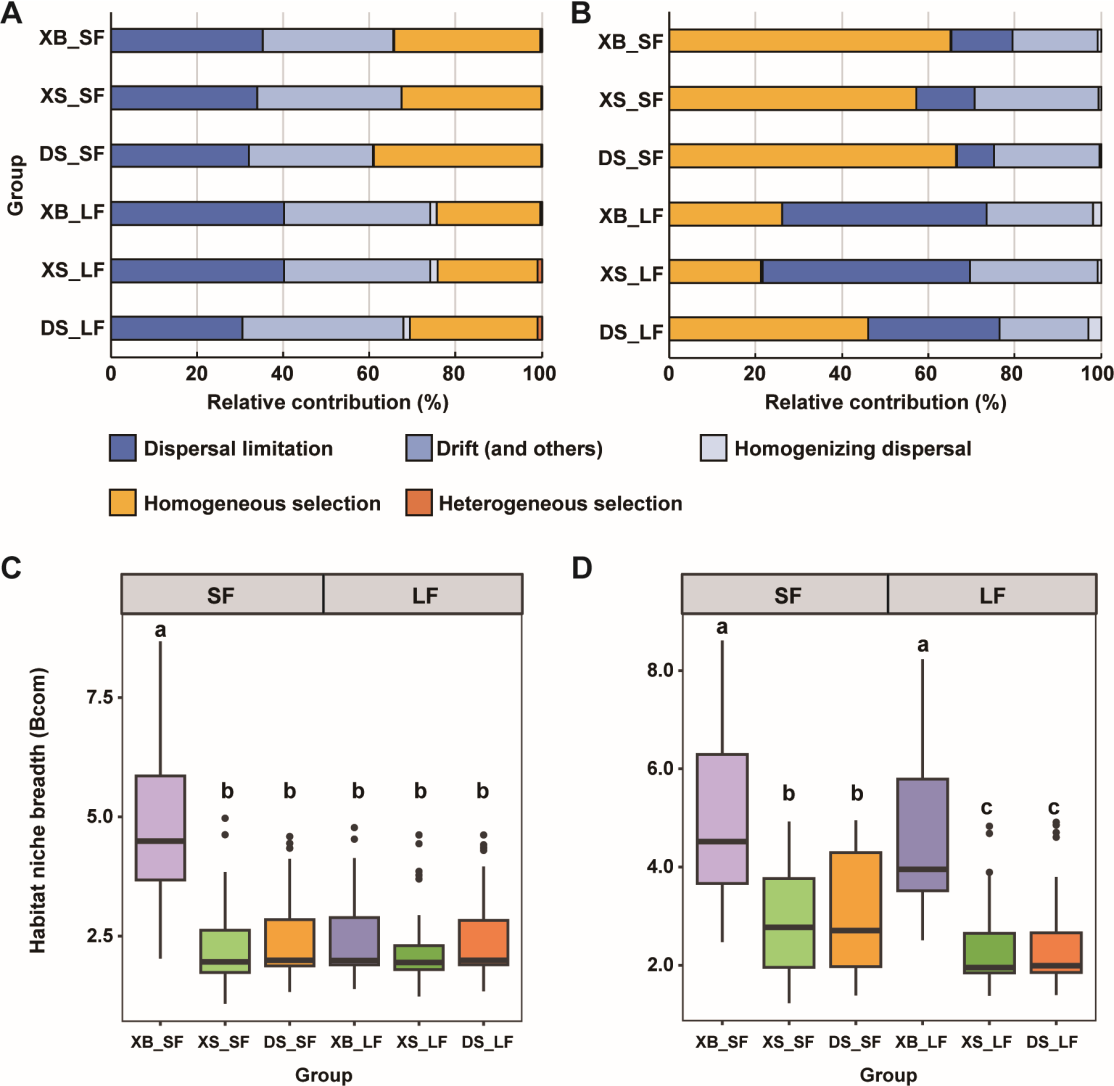
Ecological processes shaping the microbial community assembly. (**A and B**) Relative contributions of the ecological processes that determine community assembly in the bacterial (**A**) and protistan (**B**) communities at each region. (**C and D**) Comparison of mean habitat niche breadth for bacterial (**C**) and protistan (**D**) communities at each region (different letters indicate significant difference at the *P* < 0.05 level using one-way ANOVA).

We therefore employed a phylogenetic null model framework to quantify the relative importance of different community assembly processes. Null model analyses revealed that dispersal limitation, drift (and others), and homogeneous selection were the dominant mechanisms influencing the microbial communities in both seamount and coastal areas, with average relative importance values of 8.48%–47.98%, 19.72%–37.47%, and 23.12%–21.72%, respectively (Fig. 9A and B). In contrast, homogenizing dispersal and heterogeneous selection accounted for only minor fractions of community variation, with average relative importance of 0.13%–2.82% and 0.05%–0.98%, respectively (Fig. 9A and B). Homogeneous selection exerted a stronger influence on the assembly and turnover of SF microbial communities (32.41%–66.51%) than on LF communities (Fig. 9A and B). Furthermore, SF bacterial communities and both size-fraction protistan communities displayed significantly wider niche breadths in the XB seamount region than in coastal waters (Fig. 9C and D).

## DISCUSSION

### Microbial diversity and community structure in seamount and coastal regions

The α diversity of LF microorganisms was higher in the seamount region than in the coastal region, and within the seamount region, it was significantly correlated with Chl-*a* fluorescence (Fig. 2 and 7). Studies from the same cruise in the Xianbei seamount region showed that seamount topography disturbs local ocean currents and induces lee waves, thereby enhancing primary production in the euphotic zone; phytoplankton diversity and richness were higher at the Xianbei seamount than in adjacent coastal waters (23, 45). Our findings are consistent with these observations and further indicate that enhanced primary production in the euphotic zone may support higher diversity of particle-associated microorganisms in the seamount system, including PA bacteria, microeukaryotes, and zooplankton in the LF microbial community. Enhanced primary production in the euphotic zone can provide an abundant attachment substrate for PA bacteria (45), which may be one of the reasons for the high diversity of the LF bacterial community and its significant positive correlation with Chl-*a* fluorescence in the seamount region. Moreover, typical PA bacteria often have large or genetically variable genomes compared to FL bacteria, making them suitable for living in nutrient-rich and variable marine environments (46). In addition, the enhanced primary production also provided suitable food for zooplankton of LF protists in the seamount region, thus supporting higher protist diversity (47–49). This may simultaneously lead to a decrease in Chl-*a* content, which is a key pigment for photosynthesis in phytoplankton. Protists and phytoplankton may directly or indirectly compete for the same resources, such as light, nutrients, or living space, which may also lead to a negative correlation between LF protist diversity and Chl-*a* fluorescence in seamounts (Fig. 7). In summary, our findings support the notion that seamounts function as hotspots of microbial diversity, although this effect manifests in a size- and lifestyle-specific manner. Such enhancement is particularly pronounced within large-fraction, particle-associated communities, rather than being uniformly observed across all microbial groups.

The LF and SF bacterial communities of all samples were enriched in the same taxa, dominated by Proteobacteria, Cyanobacteria, and Bacteroidota (Fig. 3), which is consistent with previous studies in the SCS (50). Among protists, *Massisteria marina* was more abundant in the seamount region, which may be related to high organic matter content there (45). *M. marina* is invariably associated with detritus particles and alternates between immotile amoeboid and swimming flagellate stages, with amoeboid cells attached to particles feeding on associated bacteria and flagellated cells exploring other particles or migrating to the benthos (51, 52). Enhanced primary production in the euphotic zone of the seamount can provide rich microhabitats for *M. marina*, but the fluctuating and complex environment may also force *M. marina* to convert to the swimming flagellate stage and migrate to nearby environments, such as station XS3, which is consistent with the greater number of *M. marina* ASVs in the seamount than in the XS coastal area. Seamounts can thus not only provide special habitats for species reproduction but also provide abundant seeds for coastal regions as a “seed bank” (5), in line with previous observations of five harmful algal bloom species co-occurring in both the Xianbei seamount and XS regions (23). However, it cannot be ruled out that the relatively high abundance of *M. marina* is caused by other factors, such as sampling and sequencing methods, season, water depth, and water current.

Collectively, microbial community structure differed distinctly between the seamount and coastal regions. LEfSe analysis revealed that the seamount and adjacent coastal regions enriched distinct assemblages of bacterial and protistan taxa (Fig. 4). In general, discriminant assemblages across all regions were dominated by heterotrophic decomposers and predators, although the specific lineages fulfilling these functional roles varied substantially among regions. In the seamount region, discriminant bacterial taxa, such as Pseudomonadales and Planctomycetales, represent metabolically versatile heterotrophs adapted to particle- or surface-associated lifestyles, capable of degrading a broad range of polymeric organic substrates derived from algae and sinking particles (53, 54). These taxa likely function as key decomposers within the highly productive DCM layer overlying the seamount. The BD2-11 terrestrial group (Gemmatimonadota) consists of widely distributed yet typically low-abundance heterotrophic bacteria with versatile metabolic capabilities, which may contribute to multiple biogeochemical cycles (55). Together, these functional traits may enable these seamount-enriched bacterial lineages to thrive in the physically dynamic, resource-patchy waters above the seamount and mediate fine-scale energy and nutrient transformations.

At the protistan level, Telonemia comprises widely distributed, broad-spectrum phagotrophic flagellates that prey on bacteria and pico- to nano-phytoplankton (56). Ichthyosporea (Mesomycetozoea) are unicellular eukaryotes often associated with parasitic or symbiotic lifestyles, infecting aquatic animals such as fish (57). Thus, seamount-specific enriched taxa include metabolically flexible, particle-associated heterotrophic bacteria, as well as eukaryotic lineages involved in phagotrophic predation and host–parasite interactions. In contrast, discriminant taxa in the XS coastal region were dominated by typical particle-attached polysaccharide degraders, including Actinobacteriota and Bacteroidota (e.g., Bacteroidia, Flavobacteriaceae, and Chitinophagales), along with stalked Caulobacterales and halotolerant Salinisphaerales (58–62). These were accompanied by bacterivorous flagellates in Caecitellaceae and Kinetoplastea, which are common in highly productive, bacteria-rich coastal waters (63, 64). Together, these taxa support efficient degradation of phytoplankton- and terrestrial-derived organic matter and rapid carbon remineralization. In the DS coastal region, discriminant bacterial taxa included Marinimicrobia (SAR406 clade), Firmicutes, Xanthomonadales, *Sphingobium,* and Sphingobacteriales. These heterotrophic lineages can utilize a broad range of substrates, including relatively refractory organic matter and hydrocarbon compounds, and are frequently detected in subeuphotic or redox-structured waters (65–69). Additionally, Sagenista lineages MAST-11 and MAST-4C are ubiquitous heterotrophic flagellates that act as major bacterivores in both coastal and open-ocean systems (70).

### Reduced complexity and stability of co-occurrence networks of protist-bacterium in seamount

In this study, the potential interactions between protistan and bacterial communities in the seamount area were characterized by the lowest average clustering coefficient among the three water masses (Table 1). This demonstrates that the network was more discrete in the seamount region than in the coastal waters. This inference was further corroborated by a random-attack robustness analysis (Fig. 6A). As nodes were sequentially removed at random, the seamount network maintained consistently lower natural connectivity, whereas both coastal networks retained higher natural connectivity across the entire removal gradient, indicating reduced robustness to stochastic taxon loss in the seamount region. Studies in both marine and terrestrial environments have demonstrated that dynamic environmental heterogeneity, especially when driven by strong disturbances, can reduce the complexity of the co-occurrence patterns of microorganisms (38, 71, 72). As outlined above, the Xianbei seamount is characterized by strong environmental heterogeneity driven by intense internal waves, enhanced vertical mixing, and rapid water-mass transport (45). These processes generate rapid environmental fluctuations and impose substantial physiological stress on microbial communities, which may render microbial interactions more vulnerable to disturbance and consequently reduce the robustness and complexity of the resulting co-occurrence networks (73). Our results align with the general notion that more complex network structures tend to correspond to more stable co-occurrence patterns, whereas simple networks are often less stable (74). In the present study, the seamount network was comparatively simpler: despite having an intermediate number of edges, it exhibited the lowest average clustering coefficient and highest modularity, indicating weaker local cohesiveness and a more compartmentalized topology. Furthermore, it displayed consistently lower natural connectivity under random node removal, suggesting reduced stability. Moreover, although the seamount network harbored a great number of putative keystone taxa, the majority of these taxa functioned as connectors rather than module hubs (Fig. 6B). Keystone taxa are ecologically important because they can disproportionately affect community assembly and functional stability by mediating key biotic interactions within microbial assemblages (38). Module hubs and connectors are commonly regarded as putative keystone taxa because they contribute disproportionately to within-module cohesion and inter-module connectivity (38). Connectors act as inter-module bridges and often entail low redundancy in inter-module linkages, rendering networks dominated by such taxa particularly vulnerable to node removal. In contrast, module hubs are embedded within individual modules and typically buffered by redundant within-module connections (75). This structural reliance on connector-type keystones provides a mechanistic explanation for the observation that the seamount network, despite possessing more topologically important nodes, nevertheless exhibits the lowest overall robustness (76).

Beyond the number of keystone taxa, their taxonomic composition also varied distinctively among sampling regions. Overall, keystone taxa in both seamount and coastal networks were dominated by heterotrophic Proteobacteria (Alphaproteobacteria and Gammaproteobacteria), along with Dinoflagellata and Radiolaria. Each region additionally supported a set of habitat-specific keystone lineages. In the seamount network, Acidimicrobiia (Actinomarinales) were identified as keystone members; these are ultra-small, genome-streamlined marine actinobacteria specialized in the uptake of low-molecular-weight dissolved organic matter (77). Keystone ciliates Spirotrichea, which represent some of the most abundant planktonic micrograzers, efficiently prey on bacteria and pico- to nano-sized phytoplankton (78). In the XS coastal network, distinct keystone taxa comprised picoeukaryotic green algae (Mamiellophyceae) and heterotrophic flagellates (Katablepharidophyta) that grazed on small phytoplankton (79, 80). In the DS coastal network, unique keystone taxa included small haptophyte phytoplankton (Haptophyta, mainly Prymnesiophyceae Clade D) and heterotrophic nanoflagellates (Sagenista, mainly MAST-7) that function as bacterial grazers (70, 81). Collectively, these patterns indicate that seamount and coastal habitats are stabilized by distinct assemblages of bacterial and protistan keystone lineages, which likely underpin divergent pathways of carbon flow within the planktonic food web (82).

### Stochastic processes dominated community assembly

Microbial spatial turnover is potentially governed by various ecological mechanisms, and understanding the processes underpinning the formation of biogeographic patterns has emerged as a key factor in microbial ecology (44, 83). The DDR is among the most common biogeographic patterns; however, our findings indicate the absence of a significant DDR between the similarity of bacterial or protistan communities and geographical distance from the Xianbei seamount to coastal waters. It is widely recognized that both deterministic and stochastic processes influence DDR (84, 85); however, the relative significance of these processes varies across different habitats (86). The null model results revealed that dispersal limitation, ecological drift, and homogeneous selection processes exerted a more substantial influence than other ecological processes across all habitat types (Fig. 9A and B), leading to community differences and serving as the primary processes affecting DDR (41, 42, 83). Thus, the weak DDR suggests that geographic distance plays a limited role in driving microbial community differentiation between the seamount and coastal regions, implying that community assembly is governed by more complex processes rather than by a simple distance effect.

Estimates of niche breadth further support this interpretation. Except for LF bacteria, the habitat niche breadth of protists and SF bacteria in the seamount region was significantly broader than that in coastal waters (Fig. 9C and D). Nutrient availability and spatial heterogeneity are key axes structuring microbial niches, where more heterogeneous nutrient landscapes are often associated with broader habitat niche breadths (87). In the Xianbei seamount region, internal lee waves and intensified turbulent mixing sustain a high-productivity DCM; additionally, enhanced zooplankton activity contributes to the active vertical transport of organic matter and nutrients. Collectively, these processes generate a more complex and variable nutrient and particle environment compared to the relatively stable coastal waters, conditions that favor metabolically flexible, broad-niche taxa capable of persisting and expanding their realized niche space. This pattern is reflected in the differential enrichment of metabolically versatile taxa in the Xianbei seamount region. Such metabolic flexibility may also buffer microbial communities against local environmental filtering, enabling them to maintain compositional consistency across heterogeneous environments (43, 88), a phenomenon that may indirectly weaken the DDR of microbial communities. However, this proposed mechanistic link remains speculative and requires further empirical validation.

In the DCM water layer of the Xianbei seamount and coastal area, stochastic processes, particularly dispersal limitation and drift, appear to exert a more significant influence on bacterial communities than deterministic processes, such as homogeneous selection (Fig. 8). This observation is consistent with the findings of other studies on bacterial communities (89). However, homogeneous selection exerted a stronger influence on the assembly and turnover of SF bacterial communities (Fig. 8A), likely reflecting higher habitat homogeneity and greater hydrodynamic connectivity in this fraction. Specifically, free-living microbes in the SF fraction inhabit a well-mixed bulk water environment, whereas particle-associated taxa in the LF fraction occupy heterogeneous, patchy microhabitats with pronounced geochemical gradients. Consequently, free-living communities are more strongly governed by environmental filtering (i.e., intensified homogeneous selection), whereas particle-associated assemblages are more strongly shaped by dispersal limitation and ecological drift (89–91). This selection process results in reduced species richness and a more uniform community composition under stable environmental conditions (90, 91), which corresponds to the lower diversity indices observed in the SF than in the LF community. In contrast, stochastic processes, mainly dispersal limitation and drift, predominate in LF protist communities, whereas deterministic processes, primarily homogeneous selection, are more influential in SF protist communities (Fig. 8B). The dominant group of SF protists in the DCM water layer, from the seamount to the coastal area, was Mamiellophyceae, which are sensitive to changes in environmental conditions. Previous research has indicated that even minor disturbances in nutrient and light regimes can significantly alter the Mamiellophyceae sequence abundance (92, 93). In this study, Mamiellophyceae exhibited a high relative abundance (41.78% ± 10.59%) within the SF protist community, potentially amplifying the role of homogeneous selection. Our findings further support the hypothesis that the relative importance of stochastic and deterministic processes in shaping microbial communities is contingent on the type of microorganism involved (94).

In the context of environmental selection, inorganic nutrients are essential for microbial growth and development and are recognized as significant factors that shape the composition of microbial communities (95). The Mantel test indicated that the influence of inorganic nutrients was relatively minor when stochastic processes predominantly influenced the formation of microbial communities (Fig. 6). Although environmental factors accounted for a smaller portion of community variation, some demonstrated a strong correlation with microbial clusters of co-occurrence patterns (Fig. 7), suggesting that the effects of nutrients were more lineage-specific. The classification of microbial communities from distinctly different habitats into assemblages with specific trait combinations, based on variations in the co-occurrence or association patterns of these traits, offers new insights into the community structure and ecological functions of complex microbial communities (96, 97). The findings revealed that physical variables and nutrient conditions contributed to shifts in microbial communities in the seamount and coastal water masses.

Other unexamined environmental and biological factors, such as tides, upwelling, organic matter, and biological circadian rhythms, may also induce variations in microbial communities. In addition, we acknowledge a limitation associated with sampling design and spatial scale: our sampling stations were more than 100 km apart, so the observed community differences between seamount and coastal sites may arise from factors other than seamount-related effects, such as broader-scale geochemical gradients. While seamount-associated physical and ecological impacts have been documented to extend over tens to approximately 100 km in certain marine systems (6, 98), our sampling configuration does not allow for definitive causal inference exclusively attributed to the “seamount effect.” Future studies should employ spatially resolved sampling designs (e.g., summit-to-far-field transects with intermediate stations and replication across multiple seamounts) combined with synchronous hydrographic observations to better distinguish seamount-driven effects from regional environmental variability. Furthermore, future work should also account for temporal sampling scales and other unmeasured deterministic and stochastic factors (99).

### Conclusion

We found that the microbial diversity and community structure in the DCM of the seamount were significantly different from those of adjacent coastal areas, and we propose that these differences are likely associated with enhanced primary production and dynamic environmental heterogeneity in the seamount. Our results support the view that seamounts are hotspots of microbial diversity, and show that this pattern is size-fraction specific, with elevated bacterial and protistan diversity at the Xianbei seamount largely confined to particle-associated LF communities. The dynamic environmental heterogeneity in the seamount region may broaden habitat niche breadth for microbial communities, but may also simplify and destabilize their co-occurrence networks, leading to lower network robustness at the seamount than in coastal communities. The null model results revealed that stochastic processes (mainly dispersal limitation and drift) exerted a stronger influence on microbial community assembly, whereas homogeneous selection more strongly constrained SF communities (especially SF protists), highlighting assembly mechanisms that depend on lifestyle and size fraction. The findings of this study enhance our understanding of microbial diversity and assembly in seamounts and highlight the contrasting responses of microorganisms with different size fractions to the seamount effect. Future research should employ advanced technologies, such as metagenomics and metaproteomics, to comprehensively investigate species, genetic, and functional diversity of seamounts. This will further augment our understanding of the seamount system, thereby informing the development of effective management and conservation strategies for this unique ecosystem.

## Data Availability

The sequencing data are available on NCBI under BioProject ID PRJNA1337294.

## SUPPLEMENTAL MATERIAL

Supplemental material msphere.00162-26-s0001.docx Additional experimental details and Figures S1 to S10.

Supplemental Tables msphere.00162-26-s0002.xlsx Tables S1 to S4.

ASM does not own the copyrights to Supplemental Material that may be linked to, or accessed through, an article. The authors have granted ASM a non-exclusive, world-wide license to publish the Supplemental Material files. Please contact the corresponding author directly for reuse.
