# Supplementary material for: Composition and assembly of the microbial community within the deep chlorophyll maximum layer of the seamount and the adjacent coastal waters in the South China Sea
Source: mSphere. 2026 Jun 3;11(6):e00162-26. doi: 10.1128/msphere.00162-26 (PMC13317202; doi:10.1128/msphere.00162-26)
Supplement: Supplemental material — Additional experimental details and Figures S1 to S10. [file msphere.00162-26-s0001.docx]

## Supplemental Methods

### DNA extraction, PCR analysis and Illumina sequencing

Sequencing libraries were constructed using the MetaVX Library Preparation Kit (GENEWIZ, Inc., South Plainfield, NJ). The 25 uL PCR mixture was prepared with 2.5 uL of TransStart buffer, 2 uL of dNTPs, 1 uL of each primer, 0.5 uL of TransStart Taq DNA polymerase, and 20 ng template DNA. PCR was performed using the following program: 3 min of denaturation at 94℃, 24 cycles of 5s at 95℃, 90s of annealing at 57℃, 10s of elongation at 72℃, and a final extension at 72℃ for 5 min. Indexed adapters were added to the ends of the amplicons using a limited cycle PCR. Finally, the library was purified using the magnetic beads.

### Habitat niche breadth

To explore the relative effects of stochastic and deterministic processes on microbial communities, we calculated Levins’ niche breadth (B) index for each type of microbial community using the following formula:

$$B_{j}=\frac{1}{\sum_{i=1}^{N} {P_{ij}}^{2}}$$

where *B_j_* indicates the habitat niche breadth of ASV *_j_* in a metacommunity, *N* represents the total number of communities in each metacommunity, and *P_ij_* is the proportion of ASV *_j_* in community *i* (1, 2). A given ASV with a high B value represents a wide habitat niche breadth. The community-level B value (*Bcom*) was calculated as the average of the B values of all taxa in a given community (1, 3). A microbial community with a wide niche breadth is expected to be more metabolically flexible at the community level than one with a narrow niche breadth (1-3). The analysis was performed using the “niche.width” function within the R package “spaa”.

### References

1. Wu W, Lu HP, Sastri A, Yeh YC, Gong GC, Chou WC, Hsieh CH. 2017. Contrasting the relative importance of species sorting and dispersal limitation in shaping marine bacterial versus protist communities. The ISME Journal 12:485-494.

2. Pandit SN, Kolasa J, Cottenie K. 2009. Contrasts between habitat generalists and specialists: an empirical extension to the basic metacommunity framework. Ecology 90:2253-2262.

3. Jiao S, Yang Y, Xu Y, Zhang J, Lu Y. 2019. Balance between community assembly processes mediates species coexistence in agricultural soil microbiomes across eastern China. The ISME Journal 14:202-216.

## Supplementary Figures


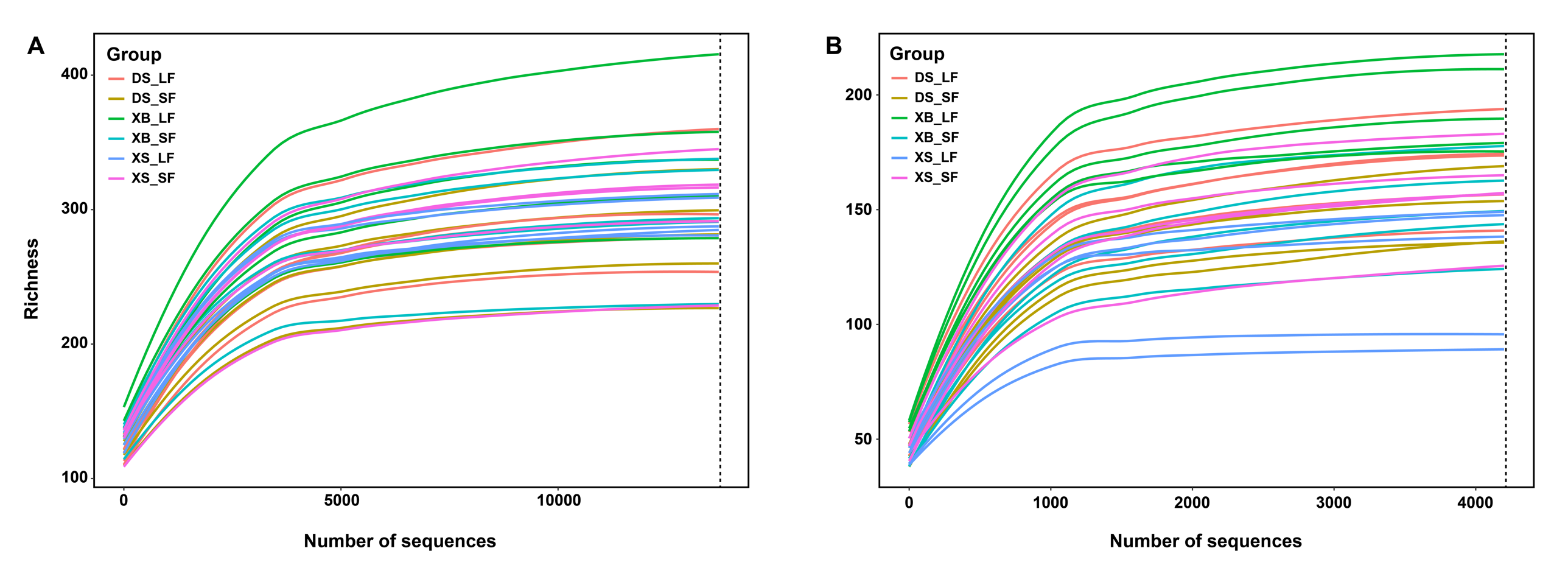


Supplementary Figure S1. Rarefaction curves of (A) bacteria and (B) protists.





Supplementary Figure S2. The concentrations of nutrients including ammonium nitrogen (NH_4_-N), nitrate nitrogen (NO_3_-N), nitrite nitrogen (NO_2_-N), and phosphate phosphorus (PO_4_-P) in the sampled seawater.


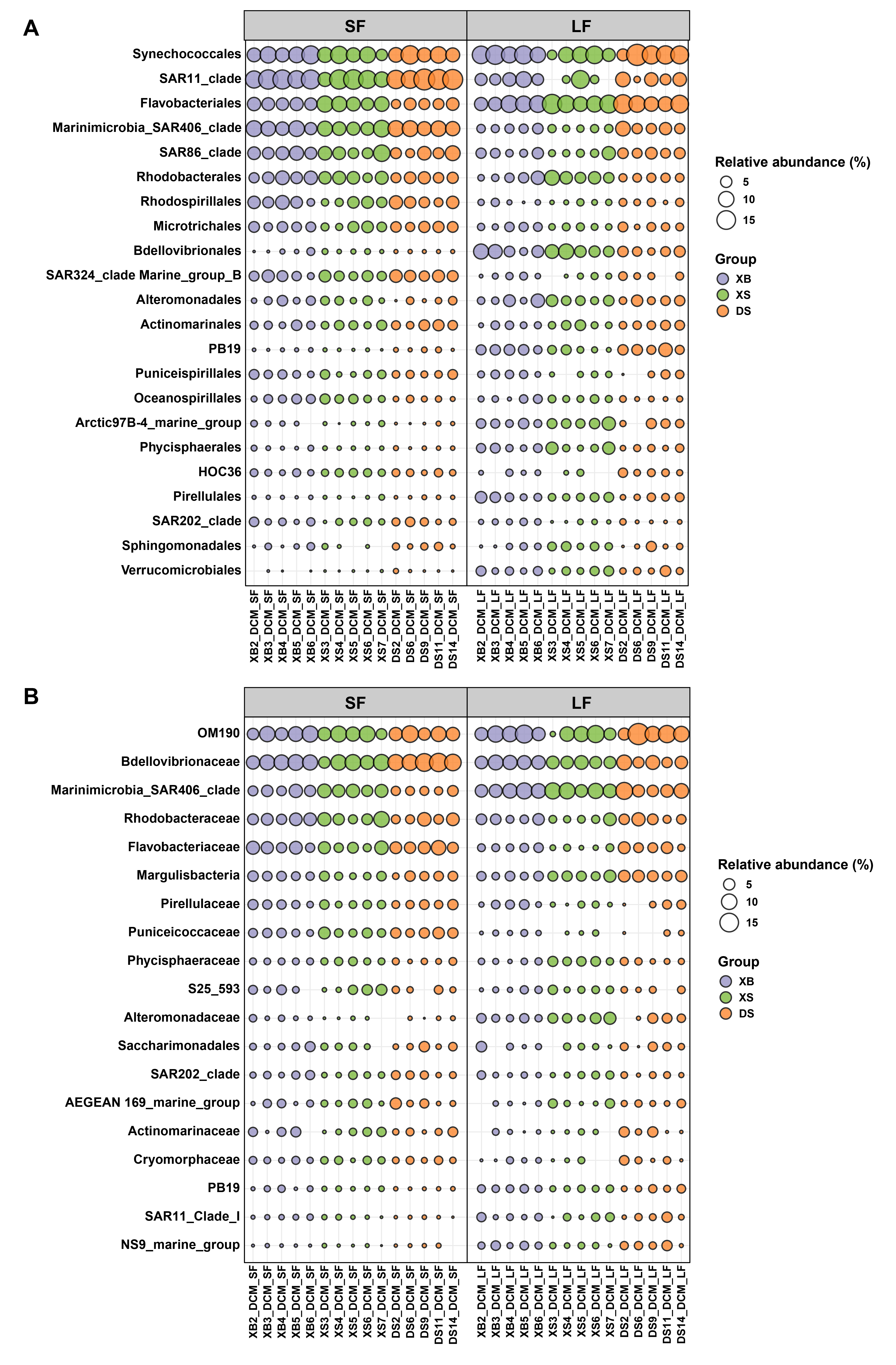


Supplementary Figure S3. The composition of bacteria in the DCM water of XB, XS, and DS at the order(A) and family(B) level.

**
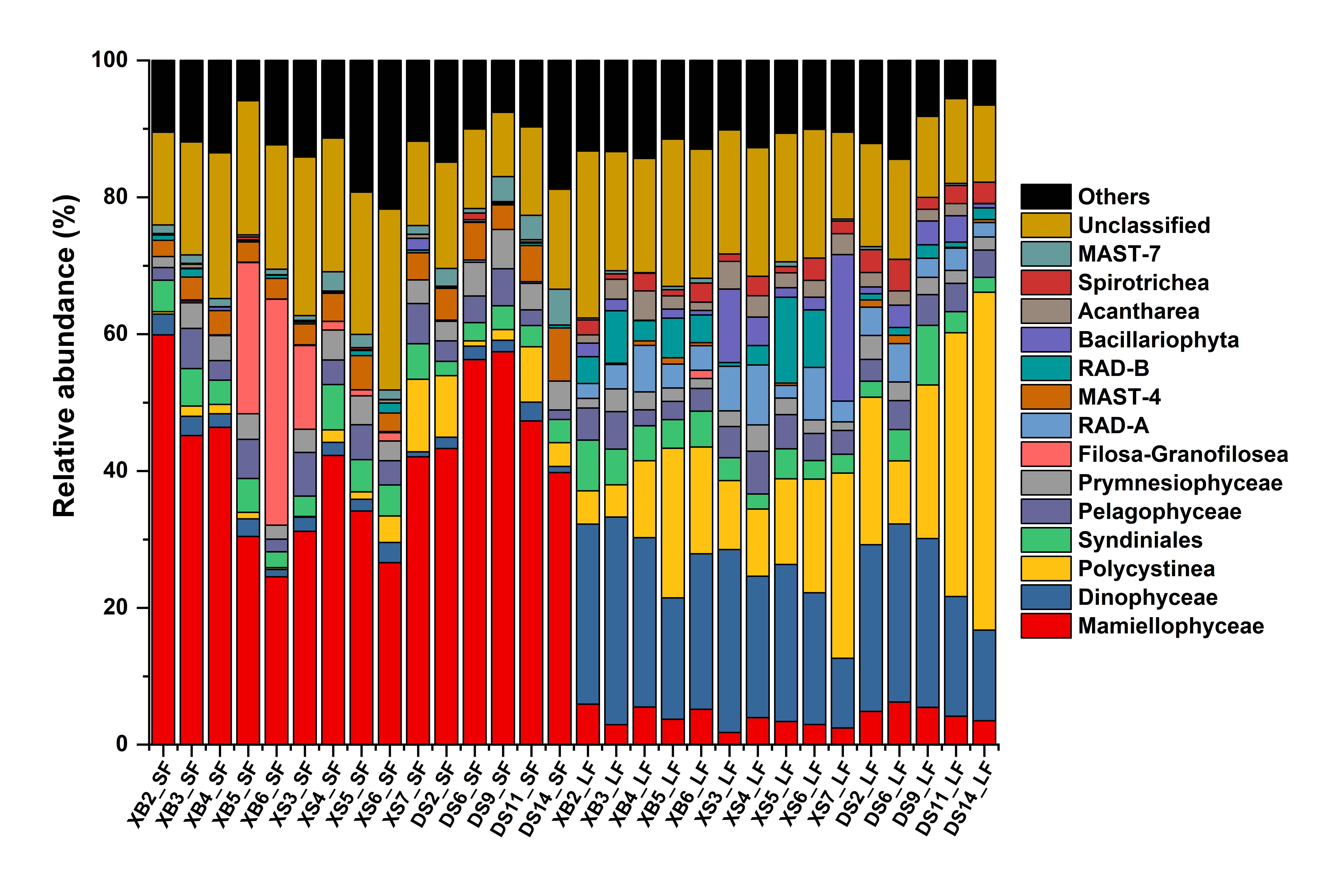
**

Supplementary Figure S4. The composition of protists in the DCM water of XB, XS, and DS at the class level.


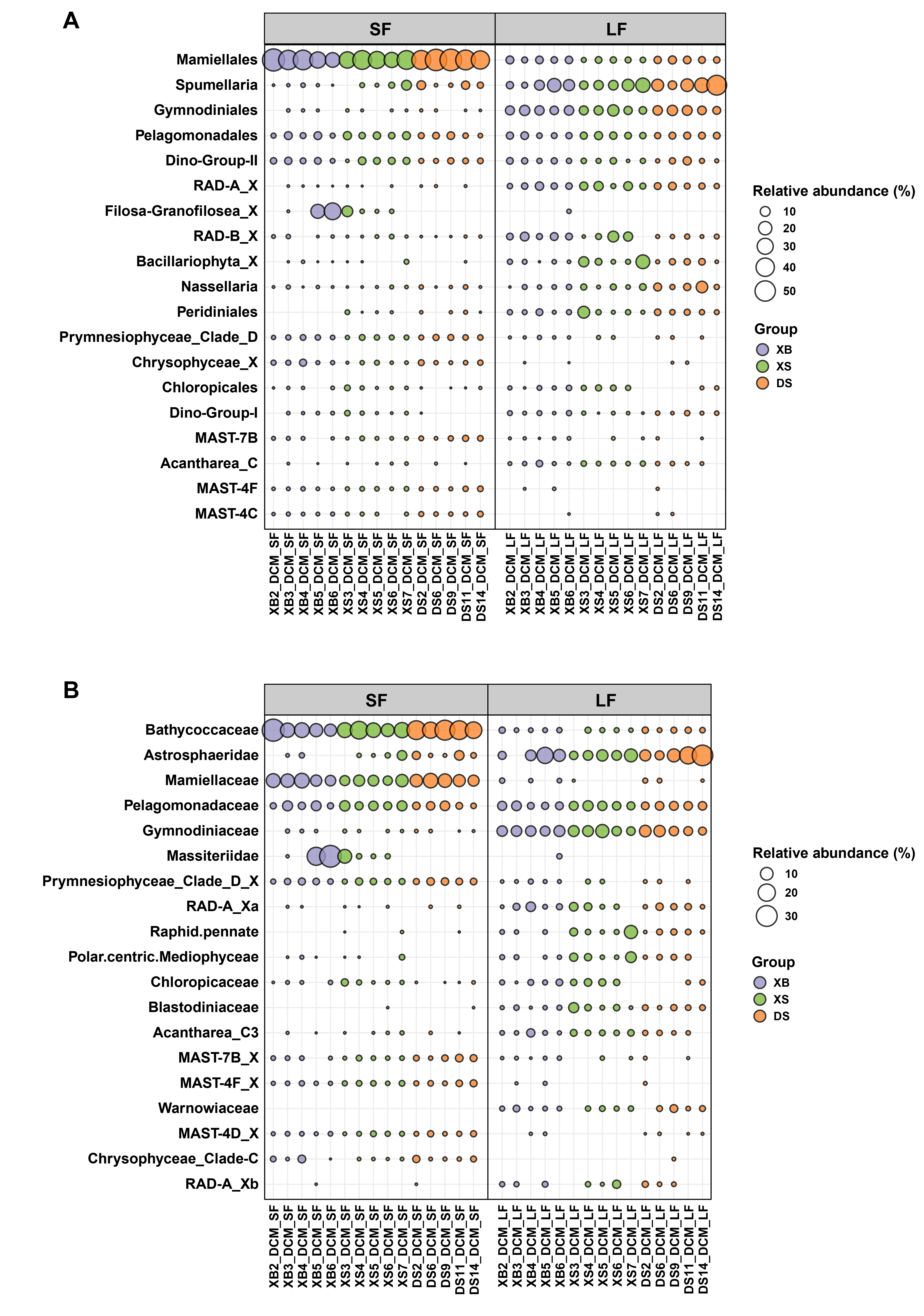


Supplementary Figure S5. The composition of protists in the DCM water of XB, XS, and DS at the order(A) and family(B) level.


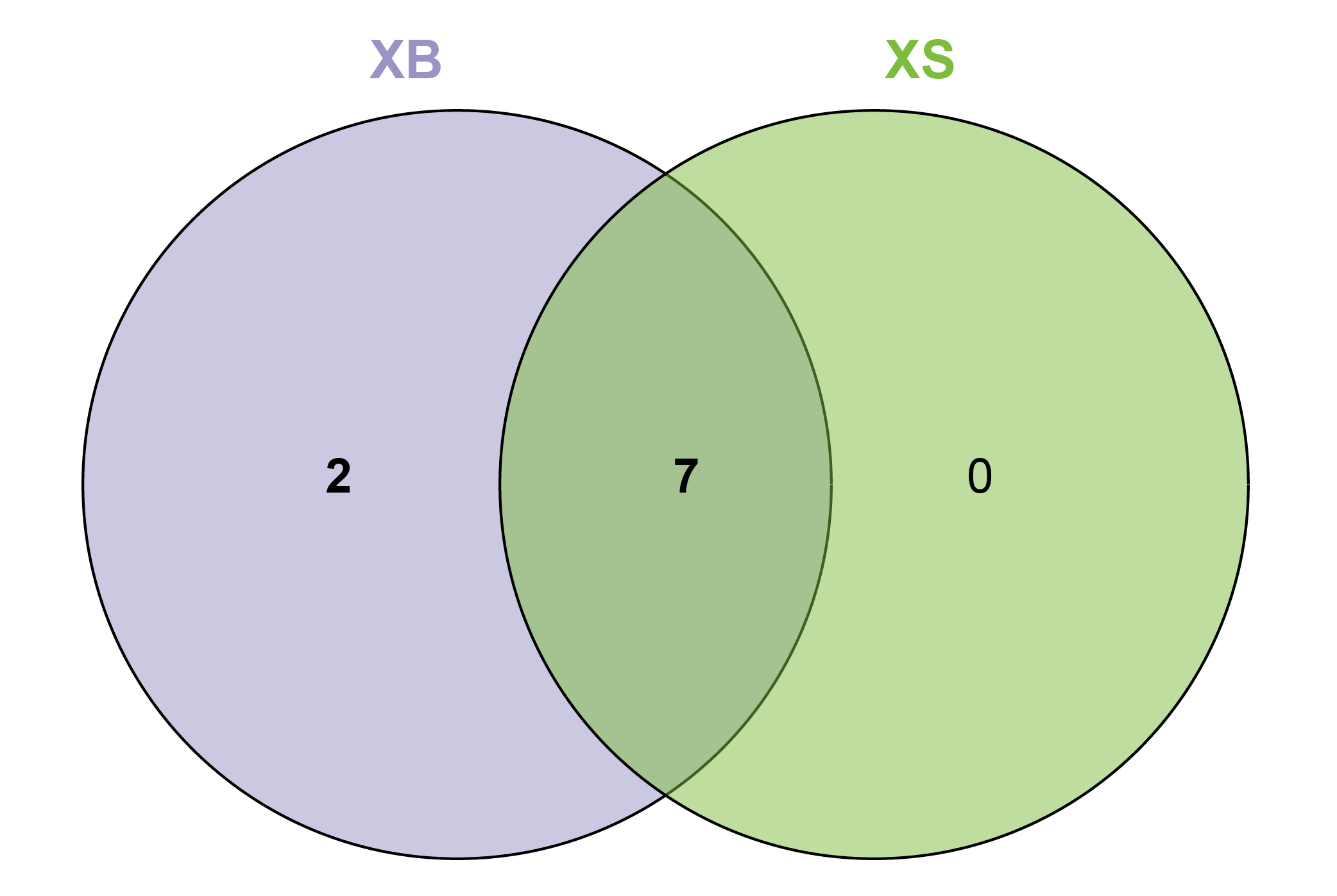


Supplementary Figure S6. Venn diagram depicted the number of unique and shared ASVs of *Massisteria marina* between XB and XS.

**
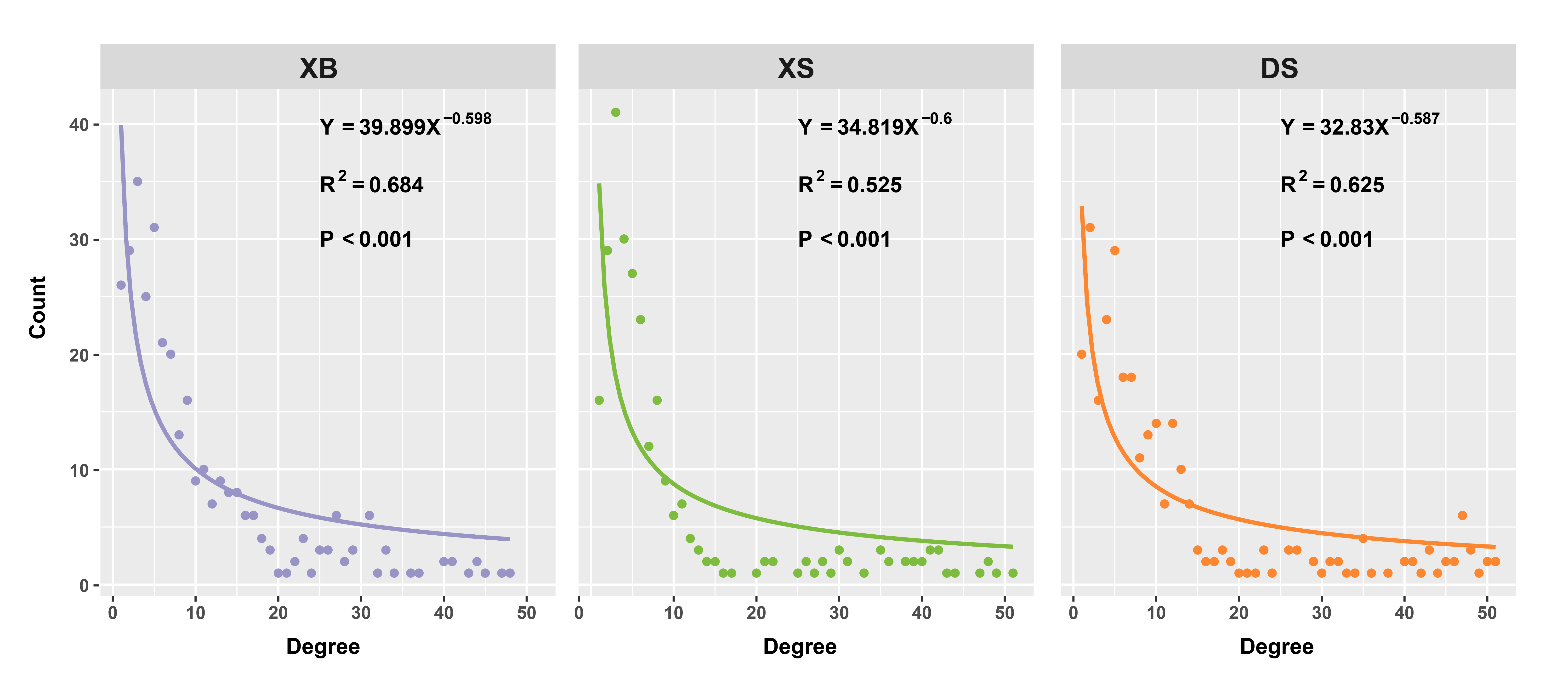
**

Supplementary Figure S7. The degree distribution of co-occurrence networks.


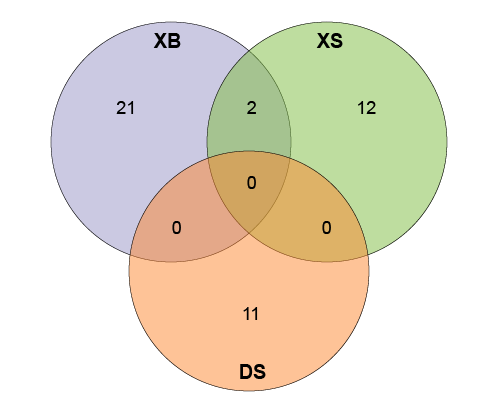


Supplementary Figure S8. Venn diagram depicted the number of unique and shared keystone nodes among XB, XS and DS networks.


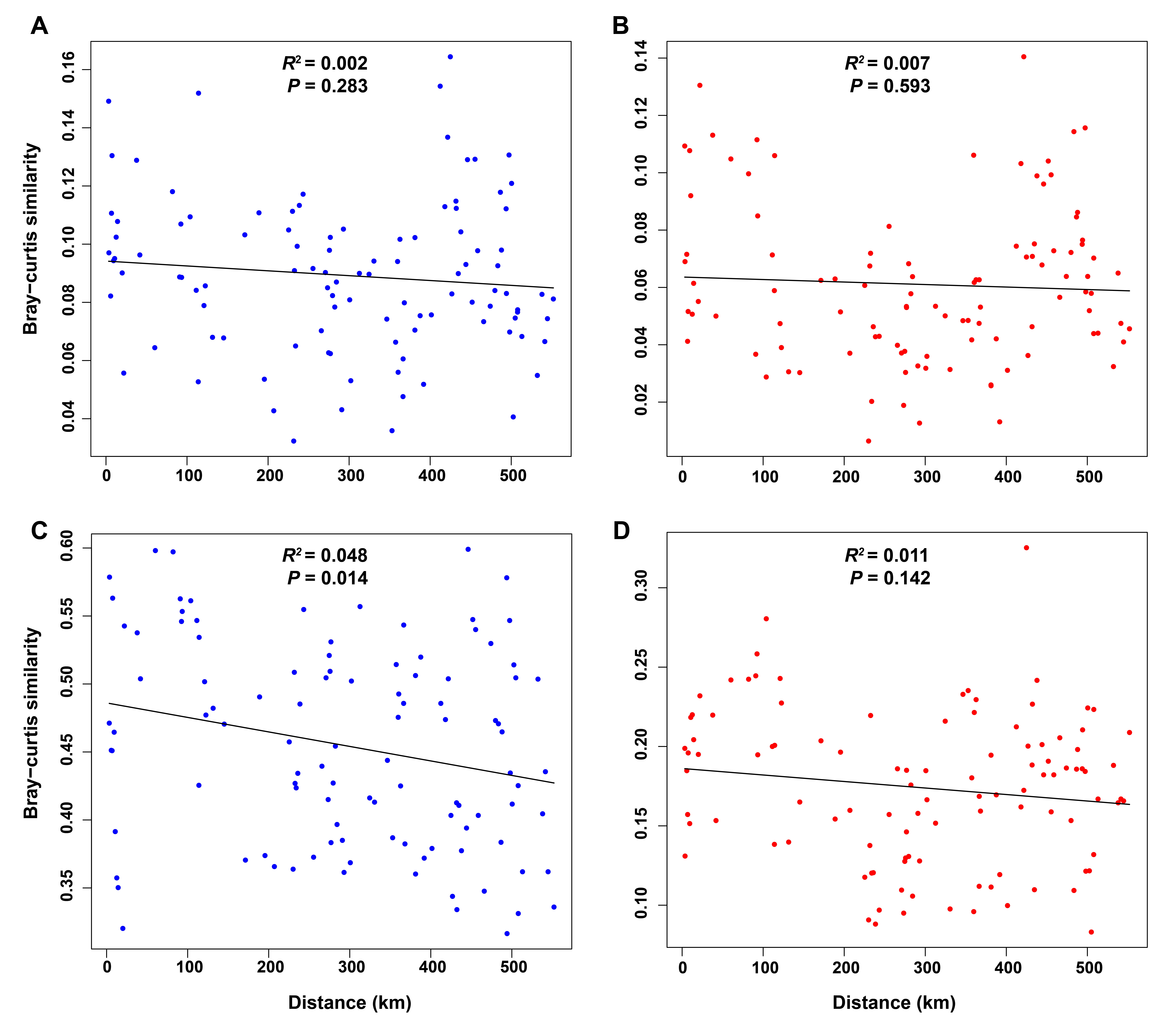


Supplementary Figure S9. Distance-decay patterns based on the Bray–Curtis similarity for the bacterial (A. SF bacteria; B. LF bacteria) and protistan (C. SF protists; D. LF protists) communities.


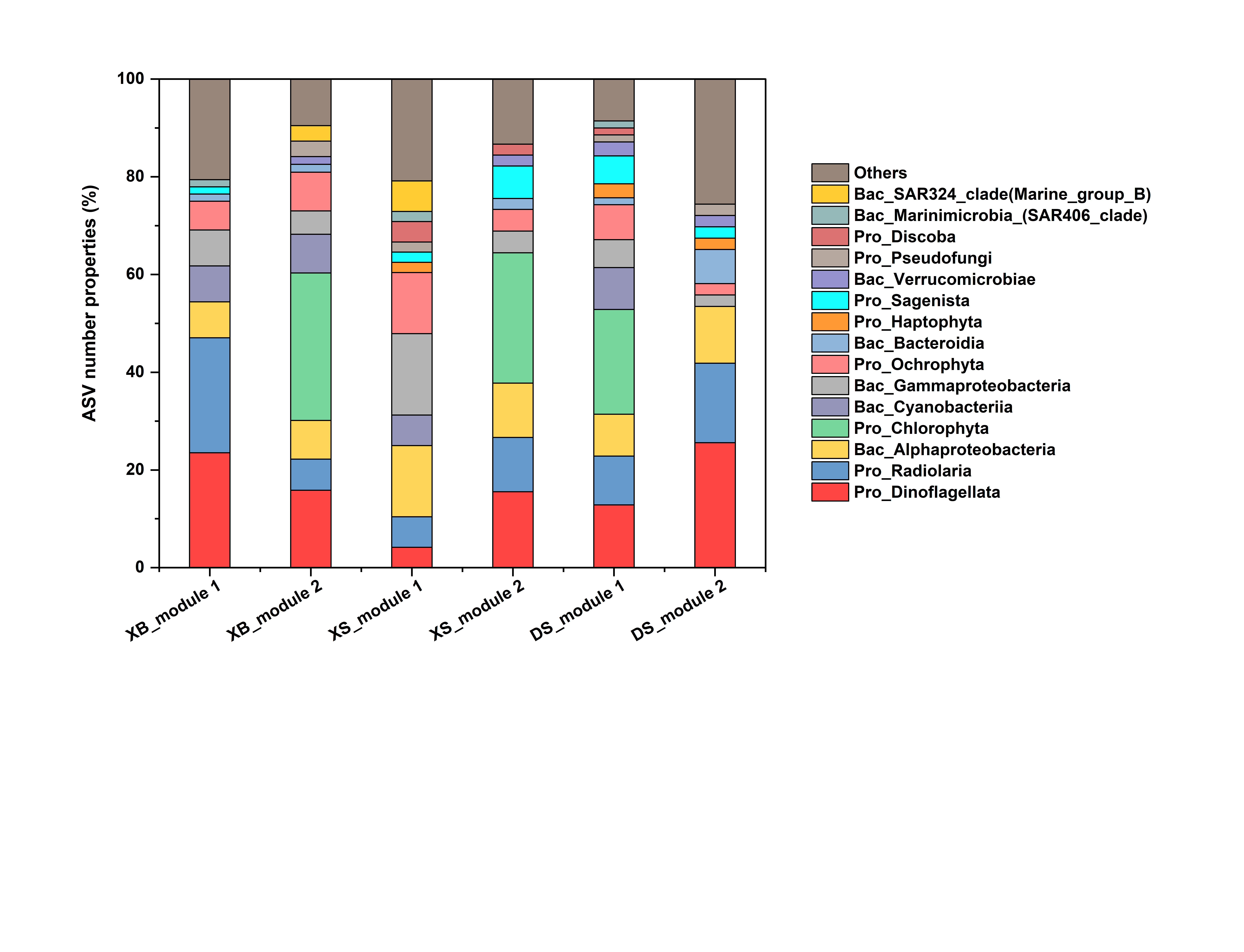


Supplementary Figure S10. The key assemblages for community composition of main modules.
